# Synthesis and Characterization of Silver-Modified Nanoporous Silica Materials for Enhanced Iodine Removal

**DOI:** 10.3390/nano14131143

**Published:** 2024-07-03

**Authors:** Ahmed Elmekawy, Qui Quach, Tarek M. Abdel-Fattah

**Affiliations:** 1 Department of Physics, Tanta University, Tanta 31527, Egypt; ahmedfelmekawy@gmail.com; 2 Applied Research Center, Thomas Jefferson National Accelerator Facility, Department of Molecular Biology and Chemistry, Christopher Newport University, Newport News, VA 23606, USA; qui.quach.13@cnu.edu

**Keywords:** silver, nanoporous, iodine removal, zeolite, natural materials

## Abstract

In aquatic environments, the presence of iodine species, including radioactive isotopes like ^129^I and I_2_, poses significant environmental and health concerns. Iodine can enter water resources from various sources, including nuclear accidents, medical procedures, and natural occurrences. To address this issue, the use of natural occurring nanoporous minerals, such as zeolitic materials, for iodine removal will be explored. This study focuses on the adsorption of iodine by silver-modified zeolites (13X-Ag, 5A-Ag, Chabazite-Ag, and Clinoptilolite-Ag) and evaluates their performance under different conditions. All materials were characterized using scanning electron microscopey (SEM), energy-dispersive X-ray spectroscopy (EDS), powdered X-ray diffraction (P-XRD), Fourier-transform infrared spectrometry (FTIR), and nitrogen adsorption studies. The results indicate that Chabazite-Ag exhibited the highest iodine adsorption capacity, with an impressive 769 mg/g, making it a viable option for iodine removal applications. 13X-Ag and 5A-Ag also demonstrated substantial adsorption capacities of 714 mg/g and 556 mg/g, respectively, though their behavior varied according to different models. In contrast, Clinoptilolite-Ag exhibited strong pH-dependent behavior, rendering it less suitable for neutral to slightly acidic conditions. Furthermore, this study explored the impact of ionic strength on iodine adsorption, revealing that Chabazite-Ag is efficient in low-salinity environments with an iodine adsorption capacity of 51.80 mg/g but less effective in saline conditions. 5A-Ag proved to be a versatile option for various water treatments, maintaining its iodine adsorption capacity across different salinity levels. In contrast, Clinoptilolite-Ag exhibited high sensitivity to ionic competition, virtually losing its iodine adsorption ability at a NaCl concentration of 0.1 M. Kinetic studies indicated that the pseudo-second-order model best describes the adsorption process, suggesting chemisorption mechanisms dominate iodine removal. Chabazite-Ag exhibited the highest initial adsorption rate with a k_2_ value of 0.002 mg g^−1^ h^−1^, emphasizing its superior adsorption capabilities. Chabazite and Clinoptilolite, naturally occurring minerals, provide eco-friendly solutions for iodine adsorption. Chabazite superior iodine removal highlights its value in critical applications and its potential for addressing pressing environmental challenges.

## 1. Introduction

In aquatic environments, the form of iodine present depends on various factors, including pH and redox conditions. At low to neutral pH and positive redox potentials, iodide (I^−^) is the dominant species in freshwater systems [1]. The long-lived radionuclide ^129^I is frequently found in contaminated groundwater and waste products from US Department of Energy facilities [2]. To mitigate the associated environmental risks, minimizing the release of ^129^I through the use of sequestration agents, also known as getters, is a potential strategy.

Getter materials should exhibit high sorption capacity for the targeted contaminant, be stable during treatment processes, and suitable for long-term waste disposal [3]. A number of materials have been evaluated for their ability to remove iodide from aqueous systems, including silver-impregnated zeolite, activated carbon, carbon fibers, and anion-exchange resins [3,4]. Among these materials, carbon fibers have been shown to have higher uptake rates compared to other options, while silver zeolites have been found to be more effective but also more expensive and potentially prone to leaching silver in acidic conditions.

The presence of iodine (I_2_) in water resources can be attributed to a variety of factors, but it has received substantial attention due to the potential dangers it may pose, especially when it is radioactive or combined with organic chemicals. Iodine is naturally occurring and can be found in seawater, which can serve as a source of iodide that can leach into drinking water aquifers and increase iodide levels in these water sources [5].

Radioactive iodine (e.g., ^129^I or ^131^I) has received specific attention among pollutants due to its harmful effects on human health and the environment. Radioactive iodine is released into the environment as a result of nuclear detonations or reactor accidents and can have negative impacts on human health and the ecosystem. Although much research has been conducted on the adsorption of iodine gas or iodine from organic solvents, there have been relatively few studies conducted on removing iodine from water at room temperature [6].

The development of materials that can effectively collect iodine from aqueous sources is essential, as radioactive iodine can be discharged into water as a direct aqueous pollutant when nuclear fission reactors are cooled with water. Major nuclear disasters, such as the Chernobyl and Fukushima accidents, serve as examples of the potential for large-scale releases of radioactive iodine into the environment, where it can be deposited in the soil and leach into water bodies [7]. The removal of radioactive iodine isotopes, such as iodine-131, iodine-125, and iodine-129, from water is crucial due to their health risks, which include thyroid cancer and other serious ailments. These isotopes often contaminate water sources through nuclear activities, medical applications, and accidental releases. Effective removal methods encompass several techniques, each suited to specific conditions. Adsorption is highly effective, particularly when using activated carbon with its large surface area and silver-impregnated materials that enhance iodine capture through the formation of silver iodide. Ion-exchange resins work by swapping radioactive iodine ions in the water with harmless, non-radioactive ions, providing a reliable means of decontamination. Chemical precipitation involves adding substances like silver nitrate that react with iodine to form insoluble compounds, which can then be filtered out of the water. Membrane filtration, including reverse osmosis and nanofiltration, leverages semi-permeable membranes to physically block and remove iodine isotopes. Advanced oxidation processes deploy strong oxidizers such as ozone or hydrogen peroxide in combination with UV light to break down iodine compounds into less harmful substances that can be subsequently removed through other methods. Each of these techniques plays a vital role in ensuring the safety and purity of water by effectively mitigating the risks posed by radioactive iodine isotopes [4,8].

Additionally, medical procedures also contribute to the presence of radioactive iodine in the environment, as radioactive isotopes are frequently used for diagnostic and therapeutic purposes. These isotopes can be excreted in the urine and end up in wastewater, leading to potential contamination of water resources [4,8].

Eliminating iodine from environmental waters can be achieved through various methods, each with distinct mechanisms and levels of effectiveness. Adsorption, utilizing materials like activated carbon and silver-exchanged zeolites, is effective due to their high surface area and strong affinity for iodine. Chemical precipitation, involving agents such as silver nitrate to form insoluble silver iodide, facilitates easy filtration [9,10,11]. Ion-exchange resins provide another efficient approach by exchanging iodine ions with other ions [12,13]. Membrane filtration techniques, including reverse osmosis and nanofiltration, effectively remove iodine by filtering water through semipermeable membranes [14,15]. Because of the unique affinity between silver and iodide ions, various types of silver-loaded solid adsorbents have been extensively utilized in the adsorption removal process of radioiodine released from nuclear reactors and other contaminates. Most of these adsorbents include zeolite, titania, activated carbon, mordenite, silica gel, and aluminum oxide, which are primarily synthesized through ion-exchange or impregnation methods. These adsorbents are nonflammable and can effectively trap organic iodine with high adsorption capacity [16,17,18,19,20,21,22,23,24,25,26,27,28,29,30,31,32,33,34,35,36,37,38,39,40,41,42,43,44,45,46,47,48,49,50,51,52,53,54,55,56,57,58,59,60]. Different types of zeolites exhibit varying physicochemical properties. Mechanical strength and acid resistance increase with a higher silica-to-alumina ratio, although an excessively high ratio can negatively impact the ion-exchange process. AgX adsorbents, in particular, show a high adsorption capacity for both organic and inorganic iodine [3,9,10,21]. Zeolites are crystalline microporous solids falling within the aluminosilicate group and the tectosilicate subgroup. Their structure comprises a three-dimensional arrangement of tetrahedral TO_4_ units (where T can be Si, Al, etc.). These materials possess pores, channels, small-molecule-sized cavities, and charge-compensating cations, rendering them highly attractive for various applications, including adsorption, catalysis, and cation exchange. In the specific context of capturing iodine species, the zeolites adsorption properties and cat ion-exchange capabilities make them exceptionally promising candidates for this purpose. Both natural and modified zeolites perform exceptionally well in removing contaminants from aqueous solutions. To address concerns about recycling and secondary pollution, zeolites can be regenerated through thermal treatment, chemical washing, or solvent extraction, effectively removing adsorbed iodine and restoring adsorption capacity. Proper management of these processes, including safe handling and disposal of desorbed iodine, ensures compliance with environmental regulations and prevents secondary pollution. Ongoing research continues to enhance the efficiency and sustainability of zeolite-based processes, further solidifying their value in wastewater remediation without causing additional environmental harm [10].

The literature has explored several zeolites with different structural types for capturing iodine species, such as mordenite (MOR structural type), NaX and NaY zeolites. In silver-exchanged mordenite, silver can exist in various oxidation states, namely as a charged Ag^+^ form and/or a reduced Ag^0^ form. They emphasized that most of these reactions are thermodynamically favorable for capturing I_2_ and CH_3_I when using mordenite-type zeolites loaded with silver [22,23,24,25,26,27,28,29,30,31].

Therefore, it is important to consider all sources of iodine in water resources and implement effective removal methods to ensure the safe use of these water sources. This includes the use of proper water treatment technologies, such as ion-exchange resins, activated carbon, and adsorption methods, to reduce the levels of iodine in water to safe levels for human consumption and other uses [3,5,32].

This study focuses on the synthesis, characterization, and comprehensive adsorption analysis of iodine using silver- modified zeolites (13X-Ag, 5A-Ag, Chabazite-Ag, and Clinoptilolite-Ag). The findings demonstrate that the materials developed hold significant potential for mitigating iodine contamination in water resources, especially in the realms of nuclear waste management and environmental protection.

## 2. Material and Methods

### 2.1. Preparation of the Sample

The present study utilized aluminum silicate in the form of zeolites (Na_2_Al_2_Si_2_O_8_·xH_2_O) as the samples. Zeolites are characterized by their porous and hydrated structure, and the presence of a variety of cations including sodium and potassium. To study the behavior of zeolites, zeolites 13X and zeolites 5A were obtained from Sigma-Aldrich Inc., St. Louis, MO, USA. In addition to synthetic zeolites, two natural zeolites were also studied. Chabazite and Clinoptilolite were chosen due to their low cost and were obtained from GSA Resources, Cortaro, AZ, USA. These natural zeolites were formed from volcanic activity and were mined in Arizona. The use of both synthetic and natural zeolites allows for a comprehensive investigation of the properties and behavior of zeolites in different forms.

Various loadings of silver were incorporated into different zeolite frameworks through ion exchange, using high-purity silver nitrate (purity > 99.9%) from Sigma-Aldrich. This process involved immersing 1.5 g of different zeolites in a 0.01 M silver nitrate solution (90 mL) at 25 °C, followed by stirring the mixture for 24 h. Subsequently, the exchanged zeolites underwent filtration and were dried overnight at 100 °C.

### 2.2. Characterization

The absorbencies of the samples were measured using Vernier spectrophotometer. The adsorbents were characterized using scanning electron microscopy (SEM, JEOL JSM-6060LV) Peabody, MA, USA), to visualize materials. Energy-dispersive X-ray spectroscopy (EDS, Thermo Scientific UltraDry, Waltham, MA, USA). In the EDS measurements, we analyzed 2 to 3 distinct areas, each 4 μm in size, within the sample to determine the elemental composition of each adsorbent. Images were taken at different magnifications and at an accelerating voltage of 15 kV. X-ray diffraction (XRD, Rigaku Miniflex II, Tokyo, Japan), Cu Kα X-ray, nickel filters) were utilized to confirm the elemental composition and crystallinity structure of adsorbents. Acquisition rates for the XRD measurements were 5° min^−1^ with a step size of 0.02°.

### 2.3. Adsorption Studies

The adsorption studies were conducted using saturated aqueous iodine I_2_ solutions (1.2 mM) as the source phase. The iodine used in this study was iodine-127. Different types of silver-exchanged zeolites materials were exposed to the saturated aqueous iodine solutions, and time-dependent UV–vis spectroscopy investigations were performed to assess the extent of iodine adsorption. The solutions were gently warmed and stirred for 12 h to 1 day to enhance the interaction between the zeolite-Ag materials and the iodine ions, as iodine is known to have low solubility in water.

#### 2.3.1. Spectrophotometry Analysis

All spectra were acquired using a UV–vis spectrophotometer with a spectral resolution of 1 nm. Aqueous solution was used as a control, and all measurements were performed using a 3.5 cm UV quartz cuvette equipped with a Teflon cap. The Vernier spectrophotometer (Beaverton, OR, USA) was carefully calibrated prior to the experiments to ensure the accuracy and reproducibility of the data.

In this study, we measured the absorption spectra of iodine-127 (*I*_2_) at a wavelength of 462 nm, tri-iodide ($I_{3}^{-}$) at a wavelength of 350.6 nm, and iodide (*I*^−^) at a wavelength of 230.1 nm [33]. The concentrations of these species were determined using calibration curves derived from their respective absorbance values. At acidic pH, iodine can react with iodide ions (if present) to form triiodide ions (($I_{3}^{-}$) as in Equations (1) and (2). At alkaline pH, iodine can form iodide (*I*^−^) and hypoiodite (*IO*^−^) as in Equation (3). However, in this study, the concentrations of *I*^−^ and ($I_{3}^{-}$ were negligible because all measurements were conducted at neutral pH, where iodine predominantly exists in its molecular form (*I*_2_).

(1) $$I_{2}+I^{-}⇌\;I_{3}^{-}$$

(2) $$\left[I_{3}^{-}\right]=K_{eq}\;{\left[I_{2}\right]}^{2}$$

(3) $$I_{2}+{2HO}^{-}⇌\;I^{-}+{IO}^{-}+H_{2}O$$

The unknown concentration of iodine in aqueous solutions was determined using a calibration curve. Six known concentrations of iodine in water were prepared and analyzed with Vernier UV–vis spectroscopy, recording spectra over 230 to 800 nm, with the peak height noted at 460 ± 2 nm, the characteristic absorption band for iodine. The calibration curve was created by plotting the peak height against the known iodine concentrations. The unknown iodine concentration in a sample solution was then determined by interpolation from this calibration curve. The iodine adsorption capacity (*q_e_*) and removal efficiency (*R*%) were calculated using the following equations:

(4) $$q_{e}=\left(\frac{C_{0}-C_{e}}{m}\;\right)V$$

(5) $$R\;\%=\left(\frac{C_{0}-C_{e}}{C_{0}}\;\right)*100$$

where *q_e_* is the iodine adsorption capacity (mg/g), *C_e_* is the concentration of iodine in the solution at equilibrium (μg/L), *C*_0_ is the initial concentration of iodine in the solution (μg/L), and m is the mass of the sorbent material (g). It is important to mention that the spectrophotometer was calibrated with a blank solution before each set of measurements, and the spectra were recorded three times for each concentration to ensure accuracy and reproducibility of the results.

The iodine isotherm study was performed to evaluate the adsorption behavior of iodine on four different types of zeolite adsorbents (13X-Ag, 5A-Ag, Chabazite-Ag, and Clinoptilolite-Ag). This study was designed to investigate the effects of different mass (50, 75, 100, 125, and 150 mg) of the silver-exchanged zeolites adsorbents on the adsorption of iodine.

A constant volume (20.00 mL) of a 1.2 mM iodine solution was added to 100 mL Nalgene bottles containing the silver-exchanged zeolites adsorbent. The bottles were then agitated at a constant rate of 50 revolutions per minute (rpm) to ensure proper mixing. The bottles were then sealed and allowed to equilibrate for 24 h at a constant temperature of 22.0 ± 1 °C. It is important to mention that the temperature was monitored and maintained using a thermocouple and a temperature controller to ensure the accuracy and reproducibility of the results. The bottles were also periodically checked to ensure that the agitation rate was consistent throughout the experiment. The equilibrated solution was then analyzed to determine the concentration of iodine remaining in solution, which was used to calculate the iodine adsorption capacity and removal efficiency as described in the previous response.

#### 2.3.2. Isotherm Study

The Langmuir and Freundlich isotherm models, which are widely used in adsorption studies, were utilized to fit the experimental uptake data. The Langmuir isotherm model, described by Equation (6), is commonly used to describe monolayer adsorption on a homogeneous adsorbent surface [34]. This equation assumes that the majority of dynamic adsorbent locations have affinities for the adsorbate, resulting in monolayer adsorption. Equation (6) was employed to describe the relationship between the equilibrium uptake (*q_e_*), maximum adsorption capacity (*q_max_*), Langmuir constant (*K_L_*), and equilibrium concentration of adsorbate in solution (*C_e_*) [34,35,36]. Specifically, *q_e_* was expressed in units of mg of adsorbate per g of adsorbent, while *q_max_* was represented in terms of mg/g. *K_L_*, on the other hand, was expressed in units of liter of adsorbent per mg of adsorbate, and *C_e_* was measured in mg/L. By plotting *C_e_*/*q_e_* against *C_e_*, a linear relationship was established, with a slope of 1/*q_max_* and an intercept of (1/*k_L_q_max_*).

(6) $$\frac{C_{e}}{q_{e}}=\frac{1}{k_{L}\;q_{max}}+\left(\frac{1}{q_{max}}\right)C_{e}$$

The Freundlich isotherm model, characterized by the Freundlich constant (*K_f_*) and the Freundlich exponent (*n*), is commonly used to describe heterogeneous adsorption processes. The value of *n*, which is always greater than one, and *K_f_* can be determined by plotting the ln *q_e_* against ln Ce and obtaining the slope and intercept, respectively Equation (7) [34].

(7) $$lnq_{e}=lnk_{f}+\left(\frac{1}{n}\right)lnC_{e}$$

Conversely, the Freundlich isotherm model, described by Equation (7), is used to model adsorption on a heterogeneous surface, where the active sites are not equally energetic.

Experimental statistics are often compared to the linear shape of Equations (6) and (7) to determine which model provides the most accurate description of adsorption by the adsorbent [35,37,38].

#### 2.3.3. pH Study

The effect of pH on the adsorption of iodine onto different types of silver-exchanged zeolites (13X-Ag, 5A-Ag, Chabazite-Ag, and Clinoptilolite-Ag) was studied. 50 mg of each of the adsorbents was added to 20.00 mL of a 1.2 mM iodine solution. The pH of the solution was then adjusted to values ranging from 2.0 to 12.0 by dropwise addition of dilute NaOH or HNO_3_.

The samples were then subjected to constant agitation (50 rpm) for 24 h at a temperature of 23.0 ± 1 °C to allow for equilibration and to ensure that the adsorbent and adsorbate were in contact for the entire duration of the experiment. The pH of the solution was continuously monitored to ensure that it remained within the desired range throughout the experiment.

This study was designed to determine the impact of pH on the adsorption efficiency of different zeolite adsorbents for iodine. The results of this study provide valuable insights into the optimal pH conditions for iodine adsorption on these adsorbents and will inform future studies on the design and optimization of adsorption processes for iodine removal.

#### 2.3.4. Ion Competition Study

The objective of this study was to determine the influence of ionic strength on the efficiency of adsorbents. A total of 0.1 g of each adsorbent was added to 20.00 mL of a 1.2 mM iodine solution, and the solutions were prepared with varying ionic strengths, specifically 0, 0.01, and 0.1 M NaCl. The mixtures were then subjected to constant agitation at 50 rotations per minute for 24 h to allow for thorough mixing. The temperature of the solutions was maintained at 22.0 ± 1 °C throughout the experiment to minimize any variations in the results.

#### 2.3.5. Kinetic Study

The adsorption kinetics of iodine on silver-exchanged zeolites adsorbents (5A-Ag, 13X-Ag, Chabazite-Ag, and Clinoptilolite-Ag) were studied using a time-dependent experimental approach. A 3.5 mL solution of 1.2 mM/L iodine in water was prepared and maintained at a temperature of 22 ± 1 °C. 50 mg of each of the four different types of silver-exchanged zeolites adsorbents was then added to separate samples of the iodine solution. The samples were exposed to the adsorbent for various time intervals (ranging from 0 to 24 h) and the concentration of iodine in the solution was determined at each time point.

The obtained data were then plotted and analyzed using two commonly used models in adsorption kinetics studies: the pseudo-first-order model (Equation (8)) and the pseudo-second-order model (Equation (9)). These models were used to determine the most appropriate and accurate representation of the experimental data, taking into account the physical and chemical characteristics of the adsorbent and the adsorbate.

It is important to note that the temperature was monitored and maintained throughout the experiment to ensure that the results were accurate and reproducible. The samples were also mixed at a constant agitation rate (50 rpm) to facilitate the mass transfer of the iodine from the solution to the adsorbent and to ensure that the experimental conditions remained consistent throughout this study. The obtained results from the adsorption kinetics study provide valuable insights into the rate and mechanism of iodine adsorption on the zeolite adsorbents.

The integrated rate law models the time variation in involved in the reaction concentrations. It is proven that Equation (8) represents the linearized integral rate law for a first-order process.

(8) $$Ln\left[C_{t}\right]=-k*t+Ln[C_{0}]$$

where *C_t_* is the concentration of solution at any time t, *C*_0_ is the original concentration at initial time and *k* (g/mg·h) is the first-order rate constant of the adsorption process

The pseudo-second-order equation is shown in Equation (9) as follows:

(9) $$\frac{1}{{[C}_{t}]}=\frac{1}{{[C}_{0}]}+k_{1}t$$

where *C_t_* is the concentration of solution at any time t, *C*_0_ is the original concentration at initial time and *k_1_* = [g/mg·h] is the second-order rate constant of the adsorption process.

#### 2.3.6. Adsorption Thermodynamics

In an effort to comprehend the thermodynamics of the adsorption process, a temperature-based study was carried out on the adsorption behavior of iodine and two different Silica-Ag adsorbents [5]. This study involved measuring the adsorption capacity of 20 mL of iodine treated with 0.1 mg of each adsorbent by gradually increasing the temperature and agitating the samples in the bottles at a constant speed of 5 rotations per minute. The experiments were performed at four temperatures of 21.0 ± 1 °C, 30 ± 1 °C, 40 ± 1 °C, and 50.0 ± 1 °C, each for a period of 24 h. The consistent temperature and agitation conditions throughout the experiment were maintained to ensure the reliability of the results.

Equilibrium thermodynamic parameters were calculated according to Lopez Delgado et al. [39] from the fraction of metal ions adsorbed:

(10) $$K_{d}=\frac{F}{1-F}$$

where *F* (the fraction of metal ions adsorbed at equilibrium) = (*F_i_* − *F_e_*)/*F_i_*

*F_i_* = initial fraction, and *F_e_* = fraction at equilibrium.

Gibbs free energy change (Δ*G^o^*), entropy change (Δ*S^o^*) and enthalpy change (Δ*H^o^*) are thermodynamic parameters and can be calculated by using Equation (11):

(11) $$\Delta G^{ο}=-RTLnK_{d}$$

where *K_d_* is the thermodynamic Langmuir constant for the adsorption process [L/mg] and *R* is the universal gas constant (8.31 J mol^−1^K^−1^), calculated using Equation (12):

(12) $$K_{d}=\frac{q_{e}}{C_{e}}$$

The entropy (Δ*S^o^*) and enthalpy (Δ*H^o^*) parameters were determined from Equation (13):

(13) $$lnK_{d}=\frac{\Delta S^{ο}}{R}-\frac{\Delta H^{ο}}{RT}$$

All adsorption studied data are included in the Supplementary Materials Figures S1–S23.

## 3. Characterization of the Adsorbent

### 3.1. XRD Characterization

**Figure 1:**
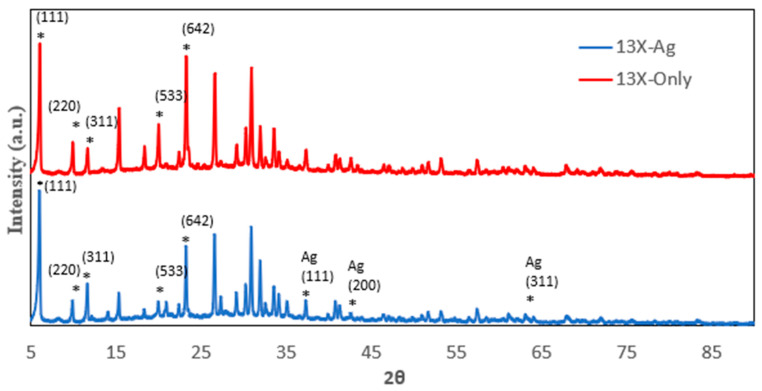
P-XRD of 13X and 13X-Ag. The asterisk (*) marked the diffraction peaks corresponding to the lattice planes.

**Figure 2:**
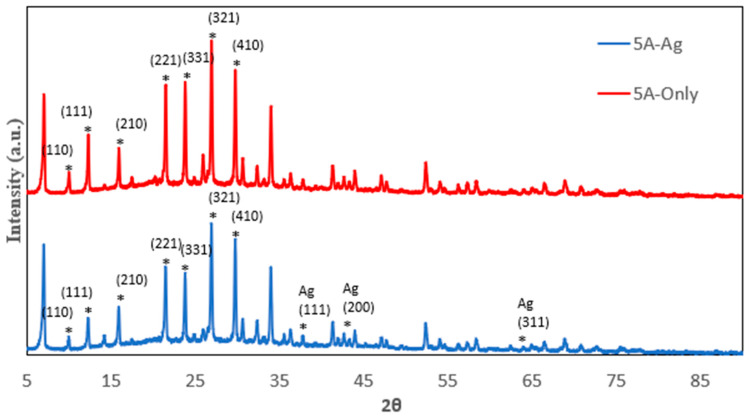
P-XRD of 5A and 5A-Ag. The asterisk (*) marked the diffraction peaks corresponding to the lattice planes.

**Figure 3:**
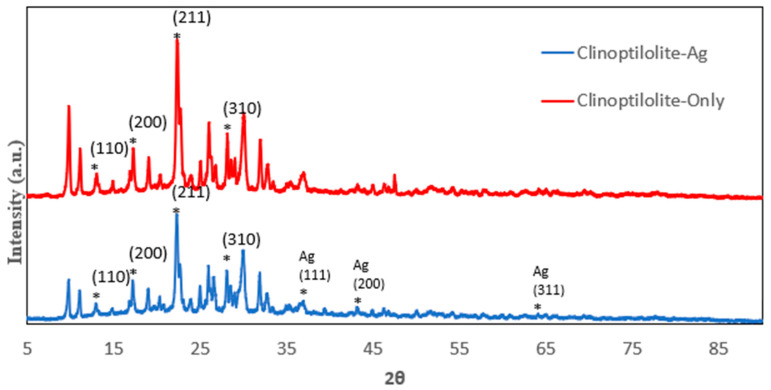
P-XRD of Clinoptilolite and Clinoptilolite-Ag. The asterisk (*) marked the diffraction peaks corresponding to the lattice planes.

**Figure 4:**
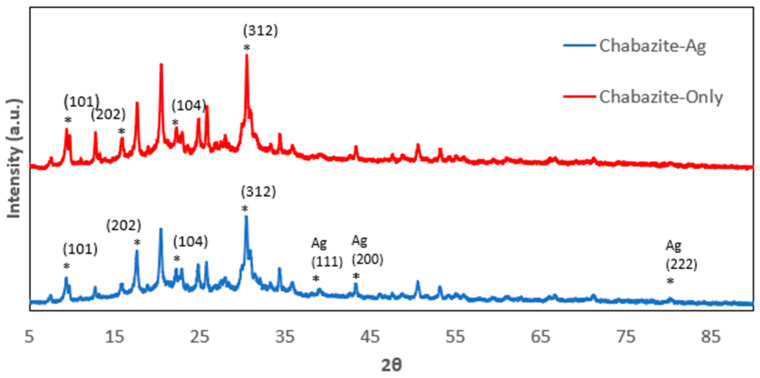
P-XRD of Chabazite and Chabazite-Ag. The asterisk (*) marked the diffraction peaks corresponding to the lattice planes.

The P-XRD spectrum of various zeolites including 13X, 5A, Chabazite and Clinoptilolite were shown on Figure 1, Figure 2, Figure 3 and Figure 4, respectively. The peaks of 13X at 6°, 10°, 12°, 15°, 20°, and 23° represented the (111), (220), (311), (533) and (642) lattice planes (JCPDS 43-0168). The 5A zeolite showed the peaks at 10°, 12°, 16°, 21°, 24°, 27°, and 29° corresponding to (110), (111), (210), (221), (331), (321), and (410) crystal planes of aluminosilicate, respectively (JCPDS 75-1151). The P-XRD of Chabazite in Figure 3 showed (101), (202), (104) and (312) lattice planes (JCPDS 01-086-1548). In Figure 4, the peaks at 13°, 17°, 22° and 28° of Clinoptilolite indicated the (110), (200), (211) and (310) lattice planes (ICSD-01-071-0962). The P-XRD silver-modified zeolites were also shown in Figure 1, Figure 2, Figure 3 and Figure 4. The peaks at 38, 44, 64 and 77 were corresponding to the (111), (200), (220), and (311) planes of Ag (JCPDS 65-2871). These results were consistent with previous publications [40,41,42,43].

### 3.2. FTIR Characterization

**Figure 5:**
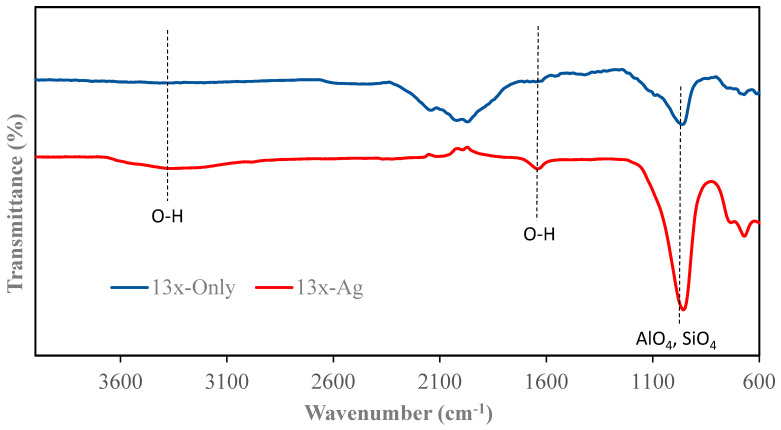
FTIR of 13X and 13X-Ag.

**Figure 6:**
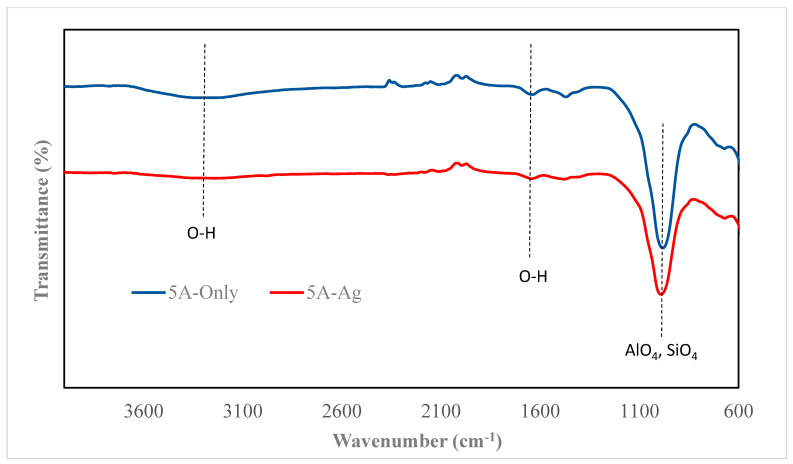
FTIR of 5A and 5A-Ag.

**Figure 7:**
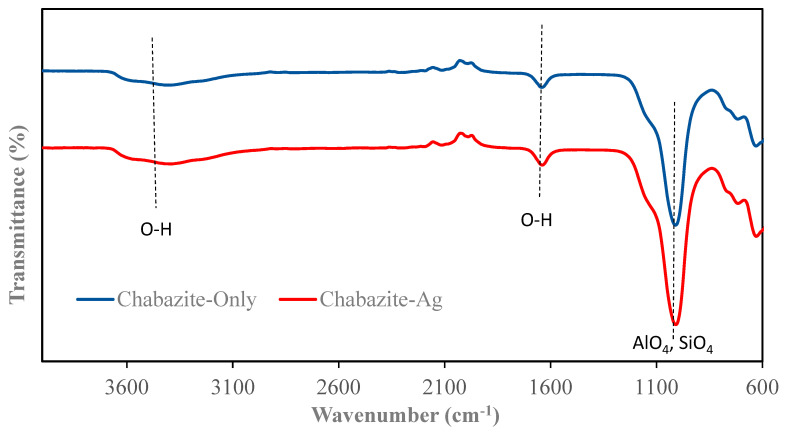
FTIR of Chabazite and Chabazite-Ag.

**Figure 8:**
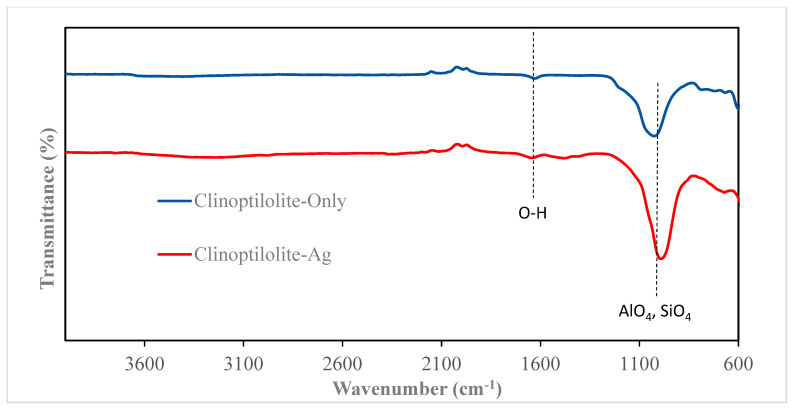
FTIR of Clinoptilolite and Clinoptilolite-Ag.

The FTIR spectra of zeolites and their silver-modified counterparts are illustrated in Figure 5, Figure 6, Figure 7 and Figure 8. All zeolite structures displayed distinct bands in the infrared spectra, specifically observed between 1010 cm^−1^ and 960 cm^−1^. These bands are attributed to the stretching vibrations of the Si-O and Al-O bonds within the zeolite framework. In the case of silver (Ag)-exchanged adsorbents, these bands exhibited slight shifts. This alteration is attributed to the incorporation of silver ions into the cavities of the zeolite structure, influencing the vibrational characteristics of the bonds involved. Additionally, the bands observed at 3450 cm^−1^ and 1643 cm^−1^ are associated with the hydroxyl groups of Si-OH and OH-Al, respectively. Notably, the FTIR spectra of the silver-modified zeolites showed no significant differences from those of the unmodified zeolites [40,41,42,43]. This consistency indicates that the synthesis process used to incorporate silver does not alter the fundamental structure or the functional groups of the zeolites.

### 3.3. SEM and EDS

**Figure 9:**
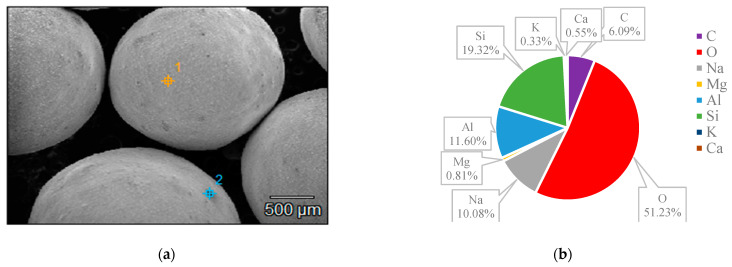
(**a**) The SEM of the 13X zeolite sample and (**b**) the EDS of the 13X zeolite sample.

**Figure 10:**
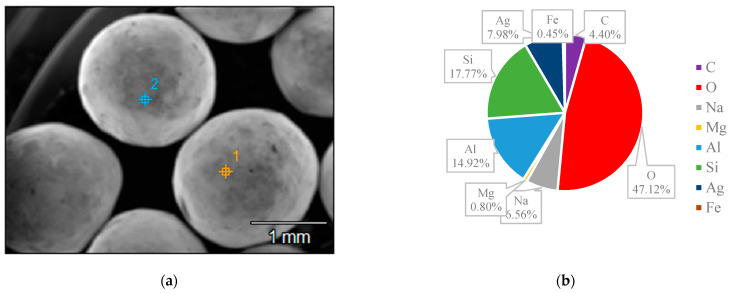
(**a**) The SEM of 13X-Ag sample and (**b**) the EDS of 13X-Ag sample.

**Figure 11:**
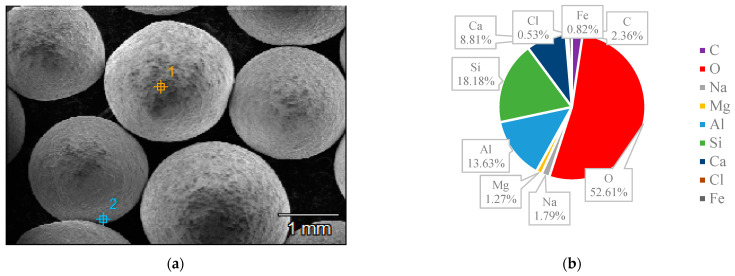
(**a**) The SEM of the 5A zeolite sample and (**b**) the EDS of the 5A zeolite sample.

**Figure 12:**
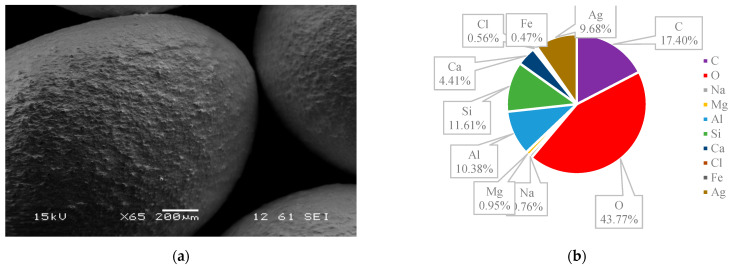
(**a**) The SEM of the 5A-Ag zeolite sample and (**b**) the EDS of the 5A-Ag zeolite sample.

**Figure 13:**
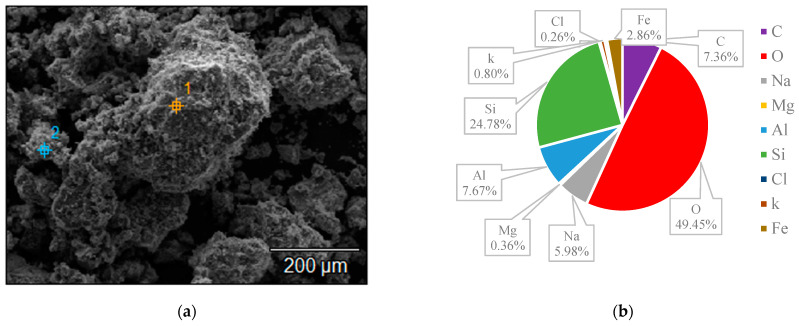
(**a**) The SEM of Chabazite sample and (**b**) the EDS of Chabazite sample.

**Figure 14:**
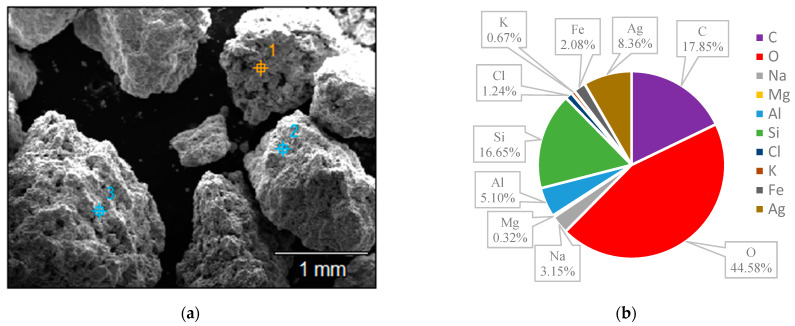
(**a**) The SEM of Chabazite-Ag sample and (**b**) the EDS of Chabazite-Ag sample.

**Figure 15:**
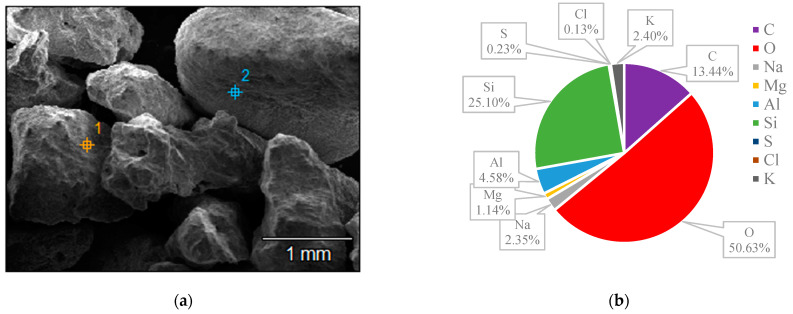
(**a**) The SEM of Clinoptilolite sample and (**b**) the EDS of Clinoptilolite sample.

**Figure 16:**
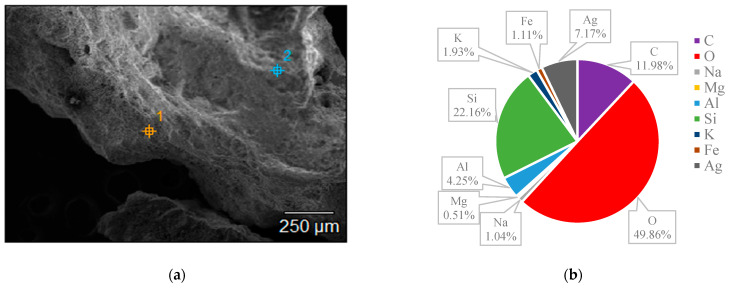
(**a**) The SEM of Clinoptilolite-Ag sample and (**b**) the EDS of Clinoptilolite-Ag sample.

The surface structures of various zeolites (13X, 5A, Chabazite, and Clinoptilolite) were compared to the silver-modified zeolites and depict from Figure 9, Figure 10, Figure 11, Figure 12, Figure 13, Figure 14, Figure 15 and Figure 16 and Tables S1–S4. The elements presented in various types of zeolites were similar. All zeolites were composed of silica (Si), aluminum (Al), calcium (Ca), oxygen (O), magnesium, and sodium (Na). Among the zeolites, Chabazite (24.78%) and Clinoptilolite (25.10%) had higher silica content than that of 13X (19.31%) and 5A (18.18%). While the aluminum content of 13X (11.60%) and 5A (13.63%) were higher than that of Chabazite (7.67%) and Clinoptilolite (4.58%). After the zeolite structure was modified by silver, the average silver content in the structure was 8.30%.

### 3.4. Surface Area

The BET surface area analysis of the zeolite samples reveals intriguing variations in their structural characteristics and potential applications (see Table 1). Pure 13X zeolite exhibits the highest BET surface area at 460 m^2^/g, emphasizing its suitability for molecular adsorption and catalysis. In comparison, pure 5A zeolite offers a slightly lower but still substantial surface area at 411 m^2^/g, indicating its potential for adsorption processes. Chabazite, in its pure form, demonstrates a BET surface area of 423 m^2^/g, highlighting its efficacy as an adsorbent material. Conversely, Clinoptilolite zeolite, with a surface area of 12 m^2^/g, may find applications where its distinct properties are advantageous. Silver exchange induces subtle changes in surface area, with 13X-Ag showing an increase to 494 m^2^/g and 5A-Ag displaying a decrease to 371 m^2^/g. Chabazite-Ag maintains a considerable surface area at 386 m^2^/g, while Clinoptilolite-Ag increased to 17 m^2^/g compared to the unmodified Clinoptilolite. In case of Clinoptilolite, silver modifications increase the micropore volume from 0.002 cm^3^/g to 0.004 cm^3^/g, contributing to a larger surface area. In addition, the original cations or other impurities present in the zeolite can block the pores. Ion exchange can remove these blocking agents, thus opening up previously inaccessible pores and increasing the surface area.

These variations underscore the structural diversity and potential applications of these zeolites, offering valuable insights for tailoring materials to specific adsorption and catalysis needs in environmental, storage, and catalytic applications.

The collective pore volume, micropore capacity, and BJH adsorption volume serve as pivotal factors influencing a zeolite aptitude for gas and molecule adsorption. Typically, pure zeolites exhibit diminished pore volumes when juxtaposed with their silver-exchanged counterparts, hinting at silver exchange’s propensity to facilitate the enlargement of internal pore networks, potentially bolstering adsorption efficiency. Of particular note, Chabazite distinguishes itself by displaying a superior overall pore volume relative to other pristine zeolites, thus accentuating its potential suitability for applications centered around adsorption.

The data concerning BJH pore size provide valuable information about the distribution of pore sizes within the zeolites. The pure Clinoptilolite zeolite presents the most extensive pore size at 126.2 Å, while among the silver-exchanged zeolites, Chabazite-Ag showcases the largest pore size at 141.2 Å. This diversity in pore dimensions underscores the versatility of zeolites and their silver-exchanged forms, allowing for customization to match specific applications, depending on the size of molecules intended for adsorption.

## 4. Adsorption Study

### 4.1. Isotherm Study

**Figure 17:**
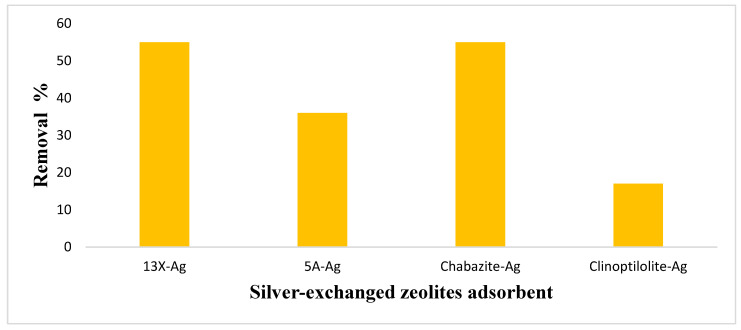
The screening iodine adsorption capacity of 13X-Ag, 5A-Ag, Chabazite-Ag, and Clinoptilolite-Ag.

In this study, we investigated the adsorption of iodine on different silver-exchanged zeolite samples: 13X-Ag, 5A-Ag, Chabazite-Ag, and Clinoptilolite-Ag. However, our investigations revealed that unmodified zeolites were ineffective in removing iodine from water. This underscores the crucial role of modification, such as the introduction of silver, in enhancing the zeolite’s capacity to adsorb iodine molecules effectively from aqueous solutions. The adsorption isotherms were analyzed using Langmuir and Freundlich models to gain insights into the iodine adsorption capacity and affinity of these materials and illustrated in Figure 17.

Langmuir Model: The Langmuir model describes adsorption on a homogenous surface with a monolayer adsorption capacity represented by *q_max_* (mg/g) and an equilibrium constant K_L_ (L/mg) reflecting the affinity of the adsorbate for the adsorbent surface. Among the silver-exchanged zeolites, Chabazite-Ag demonstrated the highest *q_max_* at 769 mg/g, indicating its excellent iodine adsorption capacity. 13X-Ag had a *q_max_* of 714 mg/g, suggesting an exceptionally high adsorption capacity, although the R-squared value (R^2^) was 0.91. The Langmuir model exhibits good correlation, although slightly lower than the fitting for the Freundlich model. 5A-Ag and Clinoptilolite-Ag displayed *q_max_* values of 556 mg/g and 192 mg/g, respectively. The K_L_ values indicate the affinity of iodine for the zeolite surface, with Chabazite-Ag having the lowest KL value (0.01 L/mg), indicating strong iodine affinity.

Freundlich Model: The Freundlich model, on the other hand, describes multilayer adsorption on heterogeneous surfaces, with K_F_ (mg/g) (L/mg)^−1/n^ representing adsorption capacity and intensity, respectively. A higher K_F_ value indicates a higher adsorption capacity, while a higher n value suggests stronger adsorbate–adsorbent interactions. Among the zeolites, Clinoptilolite-Ag exhibited the highest K_F_ value at 1485 mg/g (L/mg)^−1/n^, suggesting a robust iodine adsorption capacity. Chabazite-Ag followed with a K_F_ of 167.80 mg/g (L/mg^)−1/n^, indicating a substantial adsorption capability. 13X-Ag and 5A-Ag had K_F_ values of 8.36 mg/g (L/mg)^−1/n^ and 81.15 mg/g (L/mg^)−1/n^, respectively, reflecting their respective adsorption capacities. The n values for all samples were greater than 1, indicating favorable adsorption interactions. Among these, Clinoptilolite-Ag had the highest n value at 3.14, suggesting stronger adsorbate–adsorbent interactions.

The results of the iodine adsorption isotherm study highlight the diverse adsorption characteristics of the silver-exchanged zeolite samples (see Table 2). Chabazite-Ag exhibited the highest iodine adsorption capacity according to both Langmuir and Freundlich models, indicating its efficacy in iodine removal applications. Clinoptilolite-Ag also showed notable iodine adsorption capacity and strong interactions with iodine molecules. 13X-Ag and 5A-Ag, while having high Langmuir q_max_ values, demonstrated different adsorption behavior according to the Freundlich model, suggesting heterogeneity in their adsorption surfaces. These findings are significant for applications such as iodine removal from nuclear waste, where adsorption capacity and affinity are crucial factors. Further research could explore the practical utilization of these zeolites in iodine adsorption processes, taking into consideration their distinct adsorption behavior as elucidated by the Langmuir and Freundlich models.

### 4.2. pH Study

**Figure 18:**
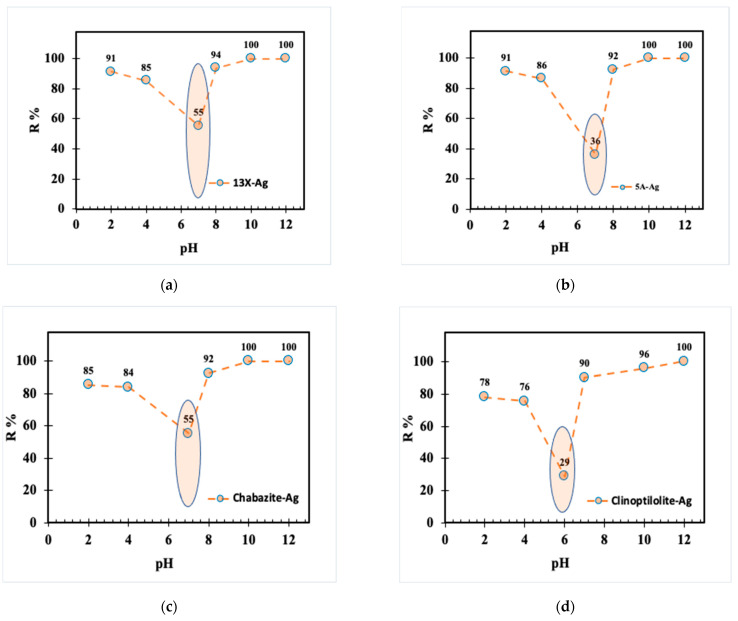
Effects of pH on iodine removal by using adsorbent (**a**) 13X-Ag, (**b**) 5A-Ag, (**c**) Chabazite-Ag, and (**d**) Clinoptilolite-Ag.

The pH study of four distinct zeolite adsorbents (13X-Ag, 5A-Ag, Chabazite-Ag, and Clinoptilolite-Ag) revealed critical insights into their performance (Figure 18). The most notable findings revolve around the pH-dependent behavior and iodine removal efficiency of these adsorbents.

The pH study of four distinct zeolite adsorbents (13X-Ag, 5A-Ag, Chabazite-Ag, and Clinoptilolite-Ag) revealed critical insights into their performance. The most notable findings revolve around the pH-dependent behavior and iodine removal efficiency of these adsorbents.

Two zeolites, 13X-Ag and 5A-Ag, consistently stood out as highly efficient in iodine removal, maintaining their effectiveness across the entire pH range tested. This characteristic makes them promising choices for applications that require reliable and robust iodine removal capabilities, irrespective of pH conditions. In contrast, Chabazite-Ag exhibited a moderate decline in iodine removal, particularly at lower pH values, indicating sensitivity to changes in pH levels. This result suggests that Chabazite-Ag may not be the optimal choice when acidic conditions prevail.

Clinoptilolite-Ag displayed the most significant pH-dependent response, with a notable decrease in iodine removal as pH levels dropped, with the most pronounced drop at pH 7. This indicates that Clinoptilolite-Ag performance for iodine removal is significantly compromised in neutral to slightly acidic conditions. These observations are essential in guiding the selection of zeolite adsorbents for practical applications. For instance, 13X-Ag and 5A-Ag are suitable when consistent and high iodine removal is required across a broad pH spectrum. In contrast, Chabazite-Ag and Clinoptilolite-Ag are more appropriate for applications in which the pH remains moderately acidic or neutral.

The decrease in iodine adsorption by zeolite at neutral pH primarily stems from the limited reactivity and weak interaction of neutral iodine molecules (I_2_) with the zeolite surface. At pH 7, iodine predominantly exists as molecular I_2_, which exhibits minimal affinity for the charged sites on the zeolite. Conversely, under acidic and alkaline conditions, iodine forms charged species such as I_3_^−^ and I^−^ (as in Equation (1)), which engage more effectively with the zeolite surface through electrostatic interactions. Acidic pH environments typically induce a positive charge on the zeolite surface, thereby enhancing attraction to negatively charged species like I_3_^−^. In contrast, alkaline conditions create a negatively charged surface, which, although not directly favorable for negatively charged I^−^, can still facilitate interactions through other mechanisms as in Equation (3). Therefore, at pH 7, the relatively neutral surface charge of the zeolite diminishes the electrostatic attraction to non-polar iodine molecules (I_2_). In contrast, both acidic and alkaline pH conditions alter the surface charge, promoting stronger interactions between zeolite and charged iodine species, thus enhancing iodine adsorption efficiency.

### 4.3. Ion Competition Study

**Figure 19:**
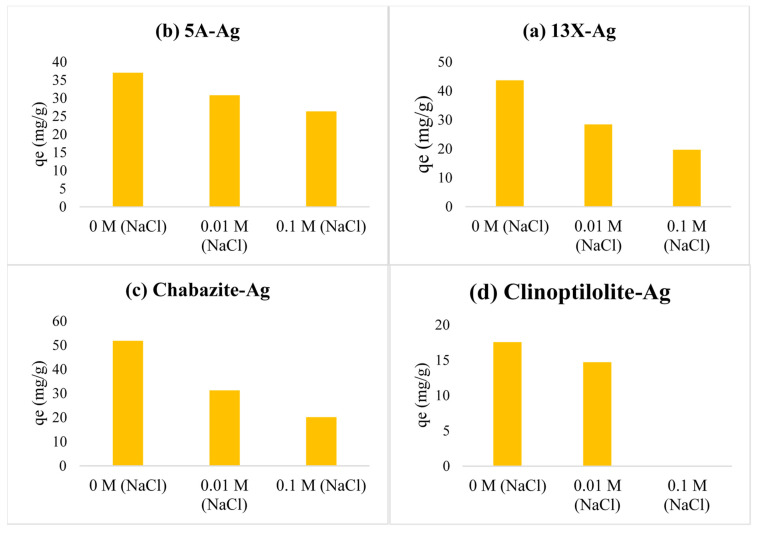
Effects of ionic strength on adsorption performance for iodine of both (**a**) 13X-Ag, (**b**) 5A-Ag, (**c**) Chabazite-Ag and (**d**) Clinoptilolite-Ag adsorbent.

Figure 19 displays the iodine adsorption capacities of four different silver-loaded zeolites 13X-Ag, 5A-Ag, Chabazite-Ag, and Clinoptilolite-Ag under varying concentrations of sodium chloride (NaCl). At a NaCl concentration of 0 M, Chabazite-Ag displayed the highest iodine adsorption capacity at 51.80 mg/g, while Clinoptilolite-Ag exhibited the lowest at 17.57 mg/g. As the NaCl concentration increased, all zeolites experienced a decline in adsorption capacities, most notably in Clinoptilolite-Ag, which virtually lost its ability to adsorb iodine at a NaCl concentration of 0.1 M.

The ionic strength competition was evident as the concentration of NaCl increased. In an environment with higher ionic strength, the competition between iodide ions and chloride ions for adsorption sites on the zeolites becomes more intense. This could explain the reduction in adsorption capacities across all zeolite types.

In the context of iodine adsorption and sensitivity to ionic strength, Chabazite-Ag demonstrates the highest efficiency at low salinity, with an impressive iodine adsorption capacity of 51.80 mg/g at 0 M NaCl, making it an excellent choice for freshwater environments. However, its effectiveness significantly diminishes at higher NaCl concentrations, limiting its suitability for saline conditions. In contrast, 5A-Ag and 13X-Ag zeolites exhibit moderate sensitivity to ionic competition, with 5A-Ag maintaining more consistent performance, making it a versatile option for various water treatment scenarios. Clinoptilolite-Ag displays a drastic reduction in adsorption efficacy, particularly at 0.1 M NaCl, rendering it unsuitable for saline applications. 5A-Ag appears as the most suitable zeolite for a broader range of applications, while Chabazite-Ag excels in low-ionic-strength conditions but may be limited in saline environments, and Clinoptilolite-Ag high sensitivity to ionic competition confines its use to specific, low-ionic-strength settings. These insights aid in zeolite selection for tailored water treatment applications.

### 4.4. Kinetic Study

In this study, we conducted a kinetic investigation of iodine adsorption on four different silver-exchanged zeolite samples: 13X-Ag, 5A-Ag, Chabazite-Ag, and Clinoptilolite-Ag. Table 3 show the two kinetic models, the pseudo-first-order and pseudo-second-order models, were employed to assess the rate at which iodine molecules were adsorbed onto these zeolite surfaces.

Pseudo-first-order Model: The pseudo-first-order model assumes that the rate of adsorption is directly proportional to the number of unoccupied adsorption sites on the surface. This model is described by the rate constant k_1_ (h^−1^). Among the silver-exchanged zeolites, Chabazite-Ag exhibited the highest k_1_ value at 0.324 h^−1^, indicating a relatively rapid initial adsorption rate. 13X-Ag, 5A-Ag, and Clinoptilolite-Ag showed k1 values of 0.288 h^−1^, 0.216^−1^, and 0.216 h^−1^, respectively. The R-squared values (R^2^) for the pseudo-first-order model were generally high, with 5A-Ag showing a perfect fit (R^2^ = 0.99), suggesting good agreement between the experimental data and the model for all samples. This indicating, the adsorption kinetic model assumes that the control of the adsorption rate depends on the diffusion of iodine on the zeolites surface.

Pseudo-second-order Model: The pseudo-second-order model posits that the adsorption process is controlled by chemisorption involving the sharing or transfer of electrons between the adsorbent and adsorbate. This model is characterized by the rate constant k_2_ (mg g^−1^ h^−1^). For all the silver-exchanged zeolites, the pseudo-second-order model provided an excellent fit to the experimental data, with R^2^ values close to 1.0. Chabazite-Ag displayed the highest k_2_ value at 0.002 mg g^−1^ h^−1^, indicating a high adsorption rate. 13X-Ag, 5A-Ag, and Clinoptilolite-Ag exhibited k_2_ values of 0.001 mg g^−1^ h^−1^, 0.001 mg g^−1^ h^−1^, and 0.003 mg g^−1^ h^−1^, respectively.

The kinetic study of iodine adsorption on the silver-exchanged zeolites reveals that the pseudo-second-order model provides an excellent description of the adsorption process for all samples, with high R^2^ values. This suggests that chemisorption mechanisms involving electron sharing or transfer play a predominant role in iodine adsorption. Chabazite-Ag demonstrated both the highest k_1_ and k_2_ values, indicating its superior initial adsorption rate and overall adsorption capacity. Conversely, 5A-Ag showed relatively lower k_1_ and k_2_ values, indicating a slower adsorption rate compared to the other zeolites. These findings are valuable for applications requiring rapid and efficient iodine adsorption, such as nuclear waste treatment. Further exploration of the practical utilization of these zeolites in iodine adsorption processes, considering their kinetic characteristics, would be a worthwhile avenue of research.

### 4.5. Temperature Effect

**Figure 20:**
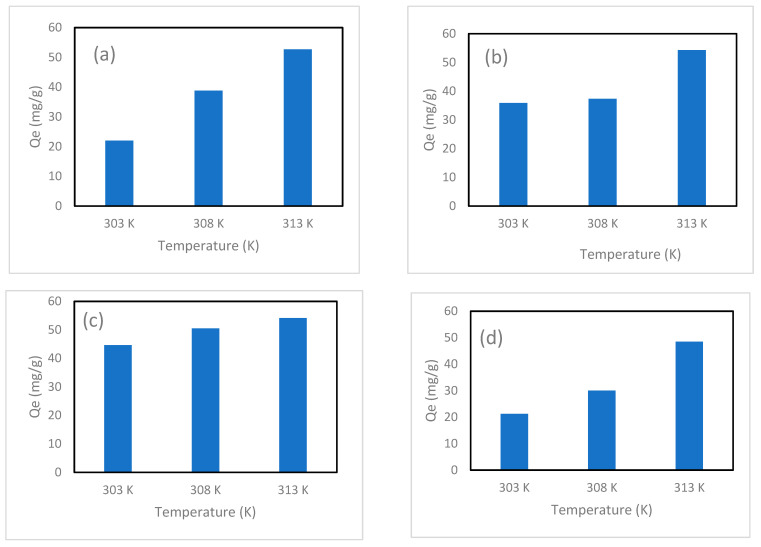
Effects of various temperatures (303 K, 308 K, 313 K) on adsorption performance of iodine for (**a**) 13X-Ag, (**b**) 5A-Ag, (**c**) Chabazite-Ag and (**d**) Clinoptilolite-Ag.

The adsorption of iodine by 13X-Ag, 5A-Ag, Chabazite-Ag and Clinoptilolite-Ag were conducted at 303 K, 308 K and 313 K and depicted in Figure 20. All composite achieved highest adsorption capacity at 313 K. At 313 K, the adsorption capacity of 5A-Ag (54.28 mg/g) was higher than that of 13X-Ag (52.68 mg/g), Chabazite-Ag (54.14 mg/g) and Clinoptilolite-Ag (48.48 mg/g). At 308 K, Chabazite-Ag exhibited an adsorption capacity of 50.48 mg/g which was higher than that of 13X-Ag (38.78 mg/g), 5A-Ag (37.32 mg/g) and Clinoptilolite-Ag (30.00 mg/g). At 303 K, Clinoptilolite-Ag achieved lowest adsorption capacity of 21.22 mg/g while Chabazite-Ag achieved highest adsorption capacity of 44.63 mg/g. The results indicated that Chabazite-Ag was ideal in iodine adsorption at various temperatures.

The high sorption capacity of zeolites at 313 K can be attributed to several factors. Firstly, the increased kinetic energy at this elevated temperature enhances the diffusion rate of iodine molecules, allowing them to reach and interact more effectively with the active sites within the zeolite structure. This heightened molecular activity leads to improved adsorption efficiency. Furthermore, higher temperatures can activate additional adsorption sites within the zeolite. This activation occurs because the energy provided at elevated temperatures can overcome the barriers to accessing these sites, which may not be available at lower temperatures. Additionally, the adsorption process of iodine onto silver-exchanged zeolites is endothermic. Therefore, higher temperatures favor this endothermic process, further enhancing the sorption capacity. The combination of increased molecular motion, activation of more adsorption sites, and the thermodynamic favorability of the endothermic process results in a significantly boosted sorption capacity of silver-exchanged zeolites at 313 K.

Through the temperature data, the van’t Hoff plots was drawn and demonstrated in the Supplementary Figures S1–S4. The negative slopes of all plots indicated that the adsorption processes are endothermic. Table 4 shows the thermodynamic parameters of 13-Ag, 5A-Ag, Chabazite-Ag, and Clinoptilolite-Ag in the adsorption of iodine at 303 K, 308 K, 313 K. All ∆G except the 303 K and 308 K trials of Clinoptilolite-Ag were negative and indicates that most reactions were spontaneous. ∆H and ∆S of 13X-Ag and Chabazite-Ag were much lower than that of 5A-Ag and Clinoptilolite-Ag. The thermodynamic parameters highly suggested that 13X-Ag and Chabazite-Ag were more energetically favorable in iodine adsorption.

In comparing the iodine adsorption capabilities of our developed materials with those from the previous studies (Table 5), it is evident that our work has introduced novel composites with notably higher capture performance. Traditional adsorbents like commercial silver-exchanged zeolite, Cu_2_O@Cu/Al-CLDH, Ag@silica gel, and Ag@zeolite demonstrated *Q_max_* values ranging from 90 to 312 mg/g, while our materials, 13X-Ag, 5A-Ag, Chabazite-Ag, and Clinoptilolite-Ag, surpassed these with remarkable *Q_max_* values of 714, 556, 769, and 192 mg/g, respectively. This significant improvement highlights the effectiveness of our materials in removing iodine compared to the established ones. The mechanical synthesis method employed in our work appears to have played a crucial role in enhancing adsorption capabilities, surpassing the performance of various zeolite silver exchange methods used in the previous studies.

### 4.6. Adsorbate, Adsorbent, and an Insightful Adsorption Mechanism

**Figure 21:**
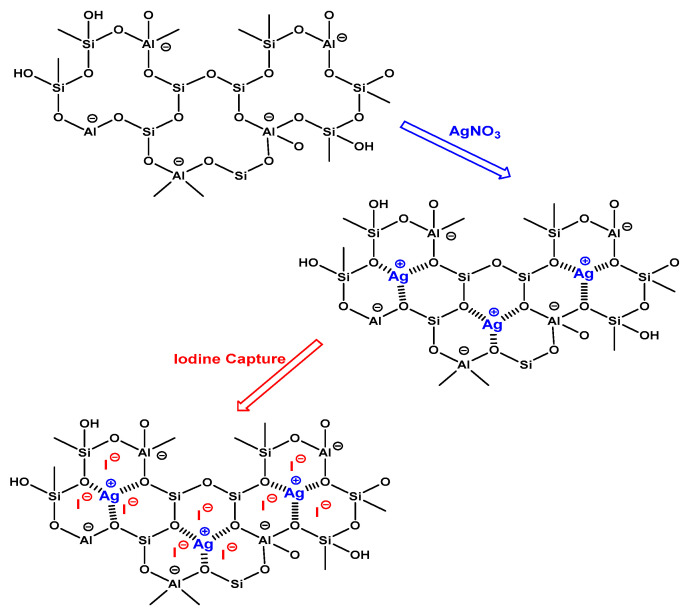
Proposed adsorption mechanism of iodine by different types of zeolite silver exchange.

To characterize the mechanisms of iodine removal using silver-exchanged zeolite, a detailed kinetic evaluation of the collected practical data was conducted [3]. This evaluation focused on mass transport and chemical reactions, primarily influenced by the contact time factor. Optimal conditions for the binding and uptake of iodine by the zeolite framework were identified. In this study, the best-fitting mechanisms were determined by applying two kinetic models: the pseudo-first-order and pseudo-second-order models, to the iodine removal data as outlined in Table 3. The main parameters used to validate the kinetic fitting included the predicted R^2^ (coefficient of determination) and qₑ (equilibrium adsorption capacity) values. The pseudo-first-order model demonstrated an average R^2^ value of 0.98 for natural zeolites (such as Chabazite and Clinoptilolite) and 0.99 for synthetic zeolites (including 13X and 5A), indicating a satisfactory fit. Conversely, the pseudo-second-order model achieved an average R^2^ value of 0.99 across all zeolites, suggesting an even better fit for the iodine adsorption onto the silver-exchanged zeolite framework. This high R^2^ value strongly suggests that the adsorption process closely aligns with chemisorption mechanisms. Notably, synthetic zeolites exhibited heterogeneity in their fit to both kinetic models. This variability underscores the complex nature of their interactions. Specifically, the interaction of iodine with the zeolite framework primarily occurs through the silver moiety, facilitating complex formation between iodine and the silver loaded onto the zeolite framework (refer to Figure 21). Therefore, the kinetic models provided valuable insights into the adsorption behavior, highlighting the effectiveness of the pseudo-second-order model in describing the adsorption process. The presence of silver in the zeolite framework significantly influences iodine adsorption, suggesting a chemisorption-dominated mechanism.

Moreover, Figure 21 illustrates the envisioned adsorption sites for zeolite silver exchange, demonstrating their ability to adsorb iodine through ion-exchange processes or electrostatic attraction [3,54]. The mechanism for zeolite silver exchange for iodine adsorption unfolds through a series of intricate steps. Initially, the zeolite framework, exemplified by 13X, 5A, Chabazite, or Clinoptilolite, harbors sodium ions (Na^+^) within its intricate three-dimensional lattice. The presence of pores, channels, molecule-sized cavities and charge compensating cations inside these materials make their properties particularly interesting for adsorption, catalysis and cation exchange. Introducing a solution containing silver ions (Ag^+^) triggers an ion-exchange phenomenon, where sodium ions are displaced by silver ions in the zeolite structure [54,55,56,57,58,59,60]. This substitution leads to the formation of a silver-exchanged zeolite, characterized by the presence of silver ions in the cationic positions of the framework. This modification primes the zeolite for iodine adsorption. The porous nature of the zeolite allows iodine molecules (I_2_) to infiltrate the framework, held in place through a combination of van der Waals forces and electrostatic attractions [54,60]. In applications such as nuclear waste management and radioactive iodine capture, zeolites demonstrate their prowess in selective ion exchange and adsorption. Furthermore, Chabazite and Clinoptilolite are notable for their natural occurrence in iodine adsorption applications, providing environmentally friendly solutions for environmental remediation. This underscores the potential of utilizing naturally occurring materials in green technologies to address environmental challenges.

Introducing a solution with silver ions (Ag^+^) triggers an ion exchange, displacing sodium ions with silver ions in the zeolite structure, forming a silver-exchanged zeolite. This modification primes the zeolite for iodine adsorption, allowing iodine molecules (I_2_) to infiltrate the framework and be held in place through a combination of van der Waals forces and electrostatic attractions. This study delves deeper into the kinetics of this process, demonstrating that the pseudo-second-order model best describes the adsorption mechanism, indicating chemisorption dominates. A key insight from our study is that natural zeolites modified with silver ions exhibit exceptionally high adsorption capacities and initial adsorption rates. This detailed analysis enhances our understanding of the adsorption mechanism beyond existing references, offering novel insights into the behavior and performance of silver-exchanged zeolites. This mechanism underscores the effectiveness of Ag-loaded zeolites in iodine capture, emphasizing their potential for use in environmental remediation applications.

In case of Chabazite and Clinoptilolite as natural zeolites modified with Ag exhibit superior adsorption performance compared to synthetic zeolites such as 13X and 5A modified with Ag due to several intrinsic characteristics. These include a more heterogeneous pore structure and wider range of pore sizes as in Table 1, which enhance their capacity to adsorb larger or differently shaped molecules. Their framework composition, containing a variety of cations and elements, creates more favorable adsorption sites as shown in Figure 9, Figure 10, Figure 11, Figure 12, Figure 13, Figure 14, Figure 15 and Figure 16, while the balance between hydrophilic and hydrophobic surface properties increases their versatility in adsorbing iodine. Additionally, natural zeolites undergo natural ion-exchange processes and often contain surface defects and mixed mineral content that contribute synergistically to their adsorption properties. The presence of hierarchical pore structures combining microporosity and mesoporosity improves mass transfer properties, allowing for more efficient adsorption of larger molecules. Furthermore, natural zeolites can exhibit better stability under certain thermal and chemical conditions, enhancing their effectiveness in various applications.

## 5. Conclusions

This study investigated the adsorption of iodine on different silver-modified zeolite samples (13X-Ag, 5A-Ag, Chabazite-Ag, and Clinoptilolite-Ag) and provided valuable insights into their performance and suitability for various applications. Langmuir and Freundlich models revealed Chabazite-Ag as the top performer, with the highest iodine adsorption capacity (769 mg/g), highlighting its efficacy in iodine removal applications, while Clinoptilolite-Ag exhibited non-significant iodine adsorption capacity (169 mg/g) and week interactions with iodine molecules. The pH-dependent study showed 13X-Ag and 5A-Ag consistently excelled in maintaining iodine removal efficiency across a broad pH spectrum, making them promising choices for applications requiring reliability. Chabazite-Ag exhibited sensitivity to lower pH values, while Clinoptilolite-Ag performance was compromised under neutral to slightly acidic conditions. Varying sodium chloride (NaCl) concentrations revealed that Chabazite-Ag excelled at low salinity, while 5A-Ag offered more consistent performance across a range of conditions. Clinoptilolite-Ag was sensitive to ionic competition, limiting its suitability for saline applications. The kinetic investigation highlighted Chabazite-Ag superior initial adsorption rate and overall adsorption capacity, beneficial for applications requiring rapid and efficient iodine removal, such as nuclear waste treatment. These findings contribute to the selection of appropriate zeolite a nanoporous adsorbents for specific applications and encourage further research into their practical utilization in iodine adsorption processes. Chabazite and Clinoptilolite, naturally occurring minerals, offer environmentally friendly solutions for iodine adsorption, Chabazite demonstrates superior efficacy in iodine removal, underscoring its higher value in applications where enhanced iodine adsorption is crucial. This emphasizes the potential of these zeolites as green technologies, aligning with the increasing importance of environmentally friendly materials in addressing pressing environmental challenges.

## Figures and Tables

**Table 1:** Adsorbent Characterization: Surface Area and Pore Volume Analysis.

Adsorbent Material	BET Surface Area m^2^/g	Single Point Adsorption Total Pore Volume of Pores cm^3^/g	t-Plot Micropore Volume cm^3^/g	BJH Adsorption cm^3^/g	BJH Volume Pore Size Å
**13X**	460	0.32	0.20	0.12	92.1
**5A**	411	0.32	0.18	0.14	101.2
**Chabazite**	423	0.43	0.19	0.25	137.9
**Clinoptilolite**	12	0.03	0.002	0.03	126.2
**13X-Ag**	494	0.34	0.22	0.12	101.7
**5A-Ag**	371	0.29	0.16	0.14	107.3
**Chabazite-Ag**	386	0.40	0.16	0.25	141.2
**Clinoptilolite-Ag**	17	0.03	0.004	0.03	118.4

**Table 2:** Comparison of Langmuir and Freundlich Model Parameters for Metal-Loaded Adsorbents 13X-Ag, 5A-Ag, Chabazite-Ag, and Clinoptilolite-Ag.

Model	Parameter	13X-Ag	5A-Ag	Chabazite-Ag	Clinoptilolite-Ag
**Langmuir**	***q_max_* (mg/g)**	714	556	769	192
	***K*_L_ (L/mg)**	0.02	0.02	0.01	0.02
	***R* ^2^**	0.91	0.94	1.00	0.90
**Freundlich**	***K_F_* (mg/g) (L/mg)^−1/n^**	60.95	81.15	167.80	1485
	***n***	2.33	3.20	3.44	3.14
	***R* ^2^**	0.99	0.85	0.92	0.65

**Table 3:** Kinetic parameters for iodine adsorption on different silver-exchanged zeolite samples.

Model	Parameter	13X-Ag	5A-Ag	Chabazite-Ag	Clinoptilolite-Ag
**Pseudo-first-order**	***k*_1_ (h^−1^)**	0.288	0.216	0.324	0.216
	***R* ^2^**	0.99	0.99	0.98	0.98
**Pseudo-second-order**	***K*_2_ (mg g^−1^ h^−1^)**	0.001	0.001	0.002	0.003
	***R* ^2^**	0.99	0.99	0.99	0.99

**Table 4:** Thermodynamic parameters for 13-Ag, 5A-Ag, Chabazite-Ag, and Clinoptilolite-Ag in the adsorption of iodine at 303 K, 308 K, 313 K.

Adsorbent	∆G (kJ mol^−1^)			∆H (kJ mol^−1^)	∆S (kJ mol^−1^·K^−1^)	R^2^
	303 K	308 K	313 K			
13X-Ag	−0.68	−1.65	−3.49	87.99	0.29	0.96
5A-Ag	−0.90	−1.15	−5.29	136.85	0.45	0.79
Chabazite-Ag	−2.54	−3.97	−5.23	84.36	0.29	0.99
Clinoptilolite-Ag	1.57	0.08	−3.43	156.29	0.51	0.95

**Table 5:** Comparison of iodine adsorption capacity of different adsorbents.

Adsorbent	Capture Performance *Q_max_* (mg/g)	References
Silica with cyclodextrin αCD, βCD, γCD and hp-βCD	435–714	[44]
Commercial silver-exchanged zeolite	312	[45]
Cu_2_O@Cu/Al-CLDH	124.6	[46]
Ag@silica gel	200	[47]
Ag@zeolite	196	[48]
Ag^0^Z	90	[49]
Ag@Mon-POF	441	[50]
H_2_CuY	450	[51]
COCuY	219	[51]
AgI-MOR	100	[52]
AgI-FAU	140	[52]
AgI-MFI	100	[52]
AgI-TON	25	[52]
Act-AgI-FAU	380	[52]
Act-AgI-MF	120	[52]
Act-AgI-TON	33	[52]
13X-Ag	714	This work
5A-Ag	556	This work
Chabazite-Ag	769	This work
Clinoptilolite-Ag	192	This work

## Data Availability

The raw data supporting the conclusions of this article will be made available by the authors on request.

## Supplementary Materials

The following supporting information can be downloaded at: https://www.mdpi.com/article/10.3390/nano14131143/s1, Figure S1: The van’t Hoff plot for the iodine adsorption of 13X-Ag at 303 K, 308 K and 313 K; Figure S2: The van’t Hoff plot for the iodine adsorption of 5A-Ag at 303 K, 308 K and 313 K; Figure S3: The van’t Hoff plot for the iodine adsorption of Chabazite-Ag at 303 K, 308 K and 313 K; Figure S4: The van’t Hoff plot for the iodine adsorption of Clinoptilolite-Ag at 303 K, 308 K and 313 K; Figure S5: Iodine Spectra UV-Vis Spectroscopy for Chabazite -Ag adsorbent; Figure S6: Iodine Spectra UV-Vis Spectroscopy for Clinoptilolite -Ag adsorbent; Figure S7: Calibration Curve for Iodine Spectra Measured with UV-Vis Spectrophotometer at 462 nm; Figure S8: Isotherm Study for 13X-Ag Measured with UV-Vis Spectrophotometer at 462 nm; Figure S9: Isotherm Study for 5a-Ag Measured with UV-Vis Spectrophotometer at 462 nm; Figure S10: Isotherm Study for chabazite-Ag Measured with UV-Vis Spectrophotometer at 462 nm; Figure S11: Isotherm Study for clinoptilolite-Ag Measured with UV-Vis Spectrophotometer at 462 nm; Figure S12: Nitrogen adsorption–desorption isotherm of 13X zeolite Adsorbent; Figure S13: Nitrogen adsorption–desorption isotherm of 13X-Ag zeolite Adsorbent; Figure S14: Nitrogen adsorption–desorption isotherm of 5A zeolite Adsorbent; Figure S15: Nitrogen adsorption–desorption isotherm of 5A-Ag zeolite Adsorbent; Figure S16: Nitrogen adsorption–desorption isotherm of Chabazite zeolite Adsorbent; Figure S17: Nitrogen adsorption–desorption isotherm of Chabazite -Ag zeolite Adsorbent; Figure S18: Nitrogen adsorption–desorption isotherm of Clinoptilolite zeolite Adsorbent; Figure S19: Nitrogen adsorption–desorption isotherm of Clinoptilolite -Ag zeolite Adsorbent; Figure S20: Adsorption kinetics of iodine on 13X-Ag adsorbent; Figure S21: Adsorption kinetics of iodine on 15A-Ag adsorbent; Figure S22: Adsorption kinetics of iodine on Chabazite-Ag adsorbent; Figure S23: Adsorption kinetics of iodine on Clinoptilolite-Ag adsorbent; Table S1: Element weight percentages by EDS for 13X and 13X-Ag adsorbent; Table S2: Element weight percentages by EDS for 5A and 5A-Ag adsorbent; Table S3: Element weight percentages by EDS for Chabazite and Chabazite-Ag adsorbent; Table S4: Element weight percentages by EDS for Clinoptilolite and Clinoptilolite -Ag adsorbent.
