# Supplementary material for: Synthesis and Characterization of Silver-Modified Nanoporous Silica Materials for Enhanced Iodine Removal
Source: Nanomaterials (Basel). 2024 Jul 3;14(13):1143. doi: 10.3390/nano14131143 (PMC11243725; doi:10.3390/nano14131143)
Supplement: Supplementary file 1 [file nanomaterials-14-01143-s001.zip › nanomaterials-3051033-supplementary.pdf]

nanomaterials

Supplementary Materials

Synthesis and Characterization of Silver-Modified Nanoporous Silica Materials for Enhanced Iodine Removal

Ahmed Elmekawy [1,2], Qui Quach [2] and Tarek M. Abdel-Fattah [2,*]

1 Department of Physics at Tanta University, Tanta 31527, Egypt; ahmedfelmekawy@gmail.com
2 Applied Research Center, Thomas Jefferson National Accelerator Facility and Department of Molecular Biology and Chemistry, Christopher Newport University, Newport News, VA 23606, USA; qui.quach.13@cnu.edu
* Correspondence: fattah@cnu.edu

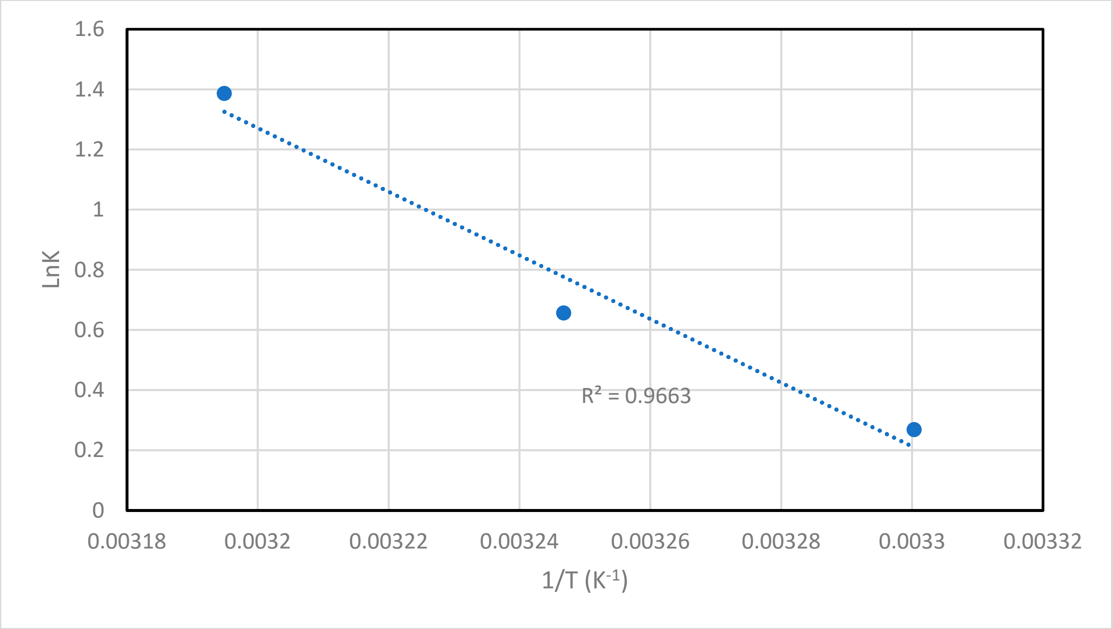

Figure S1. The van't Hoff plot for the iodine adsorption of 13X-Ag at 303 K, 308 K and 313 K.

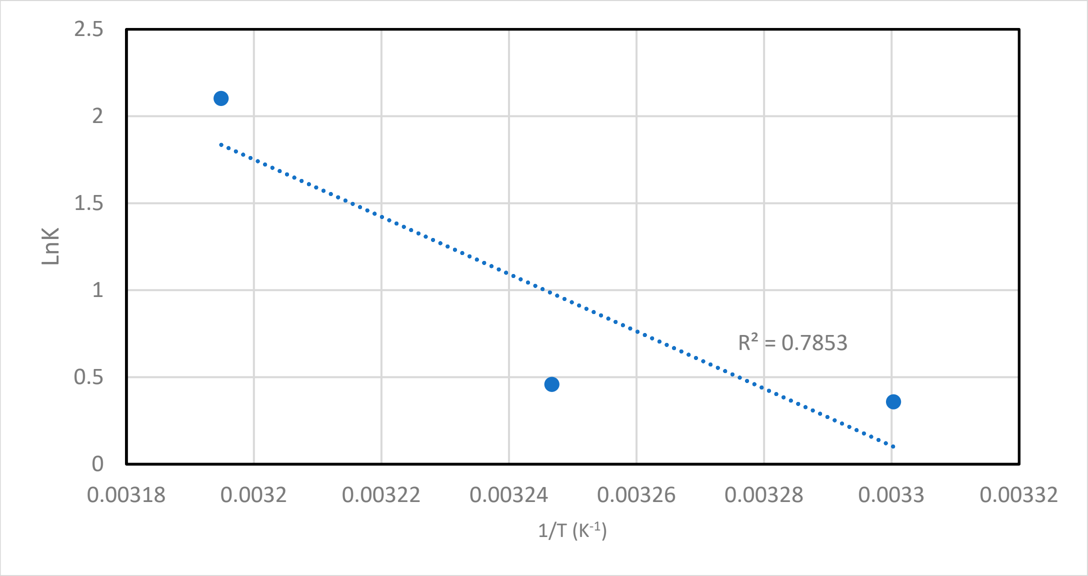

Figure S2. The van't Hoff plot for the iodine adsorption of 5A-Ag at 303 K, 308 K and 313 K.

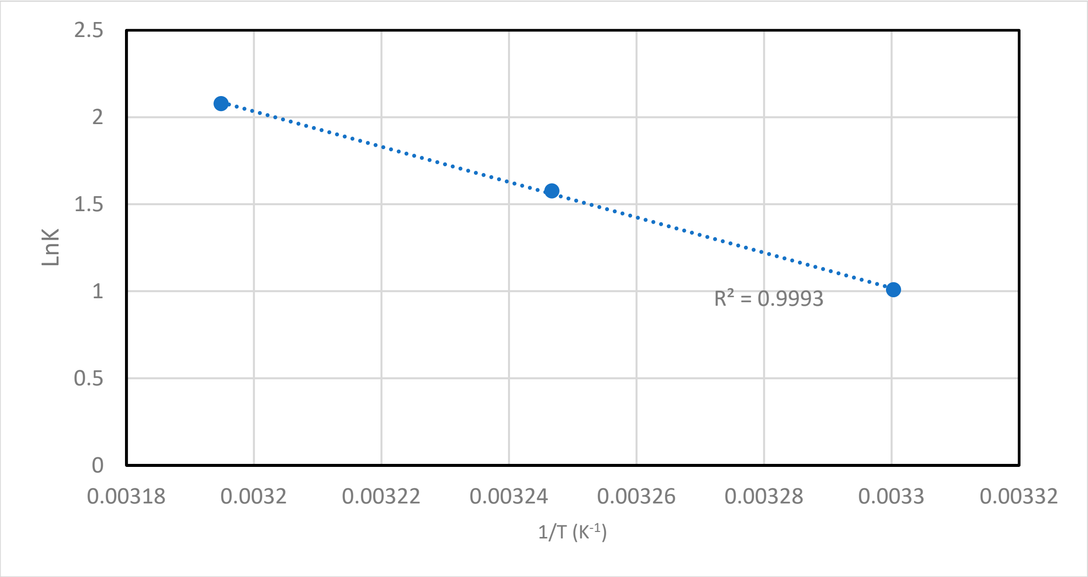

Figure S3. The van't Hoff plot for the iodine adsorption of Chabazite-Ag at 303 K, 308 K and 313 K.

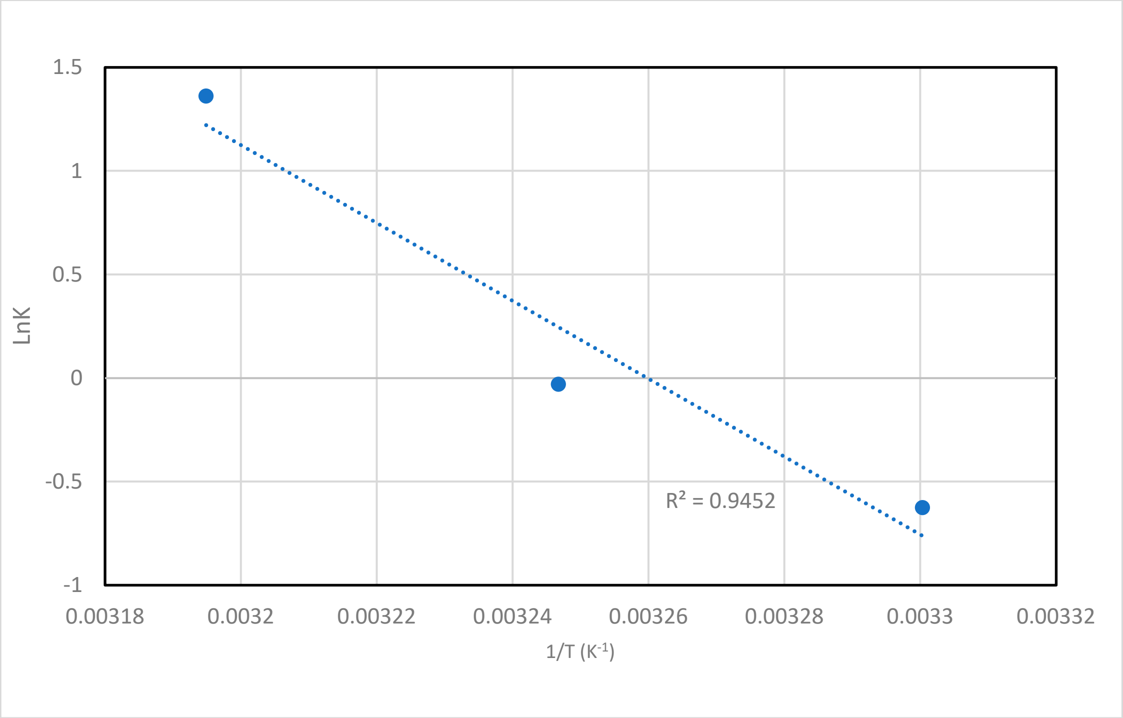

Figure S4. The van't Hoff plot for the iodine adsorption of Clinoptilolite-Ag at 303 K, 308 K and 313 K.

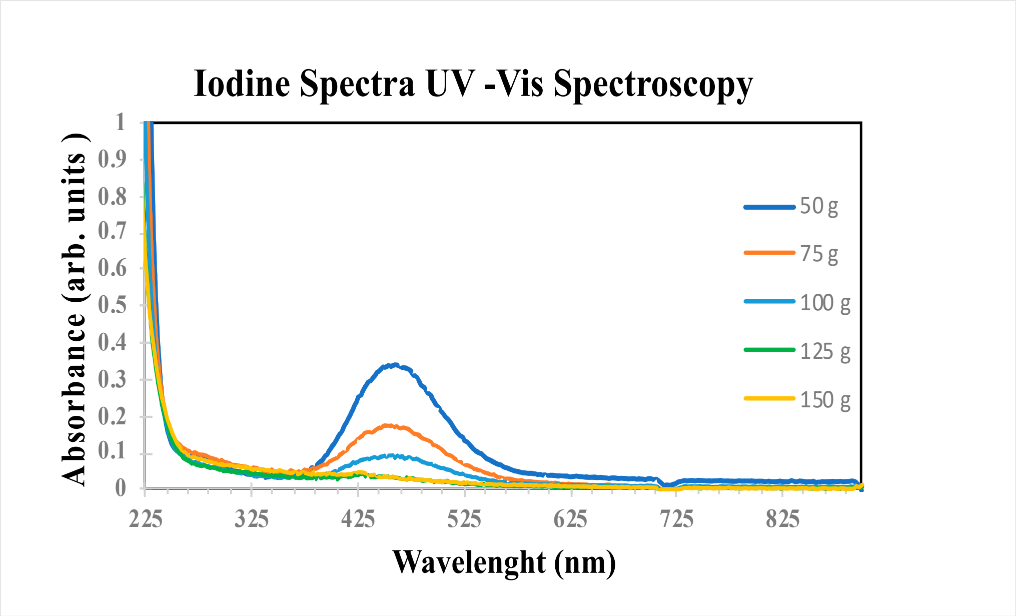

Figure S5. Iodine Spectra UV-Vis Spectroscopy for Chabazite -Ag adsorbent.

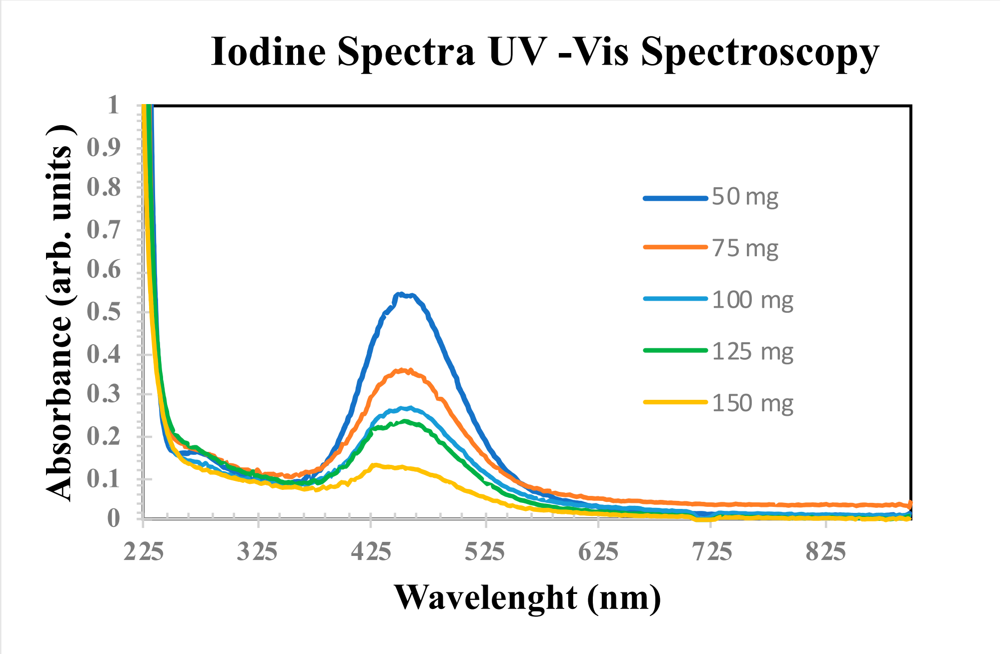

Figure S6. Iodine Spectra UV-Vis Spectroscopy for Clinoptilolite -Ag adsorbent.

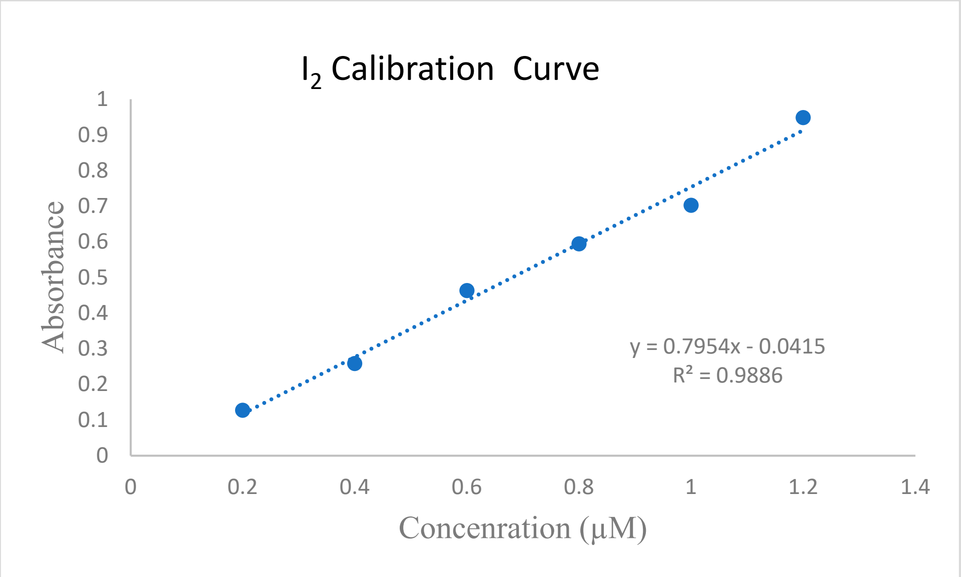

Figure S7. Calibration Curve for Iodine Spectra Measured with UV-Vis Spectrophotometer at 462 nm.

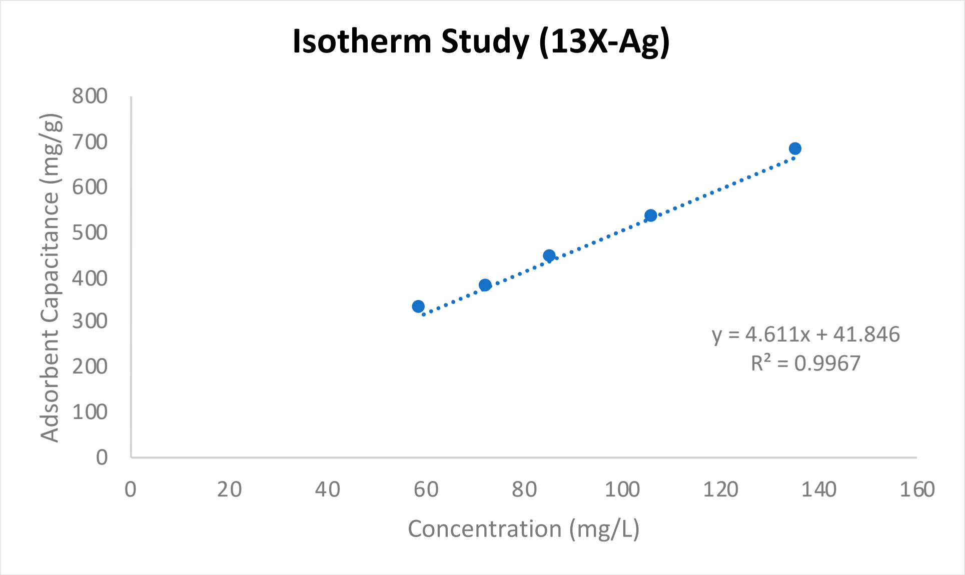

Figure S8. Isotherm Study for 13X-Ag Measured with UV-Vis Spectrophotometer at 462 nm.

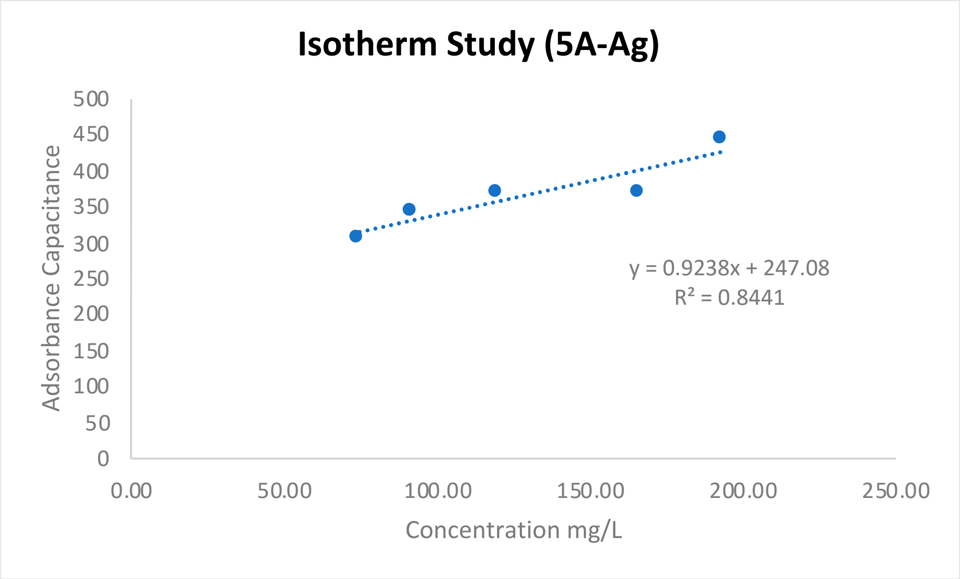

Figure S9. Isotherm Study for 5a-Ag Measured with UV-Vis Spectrophotometer at 462 nm.

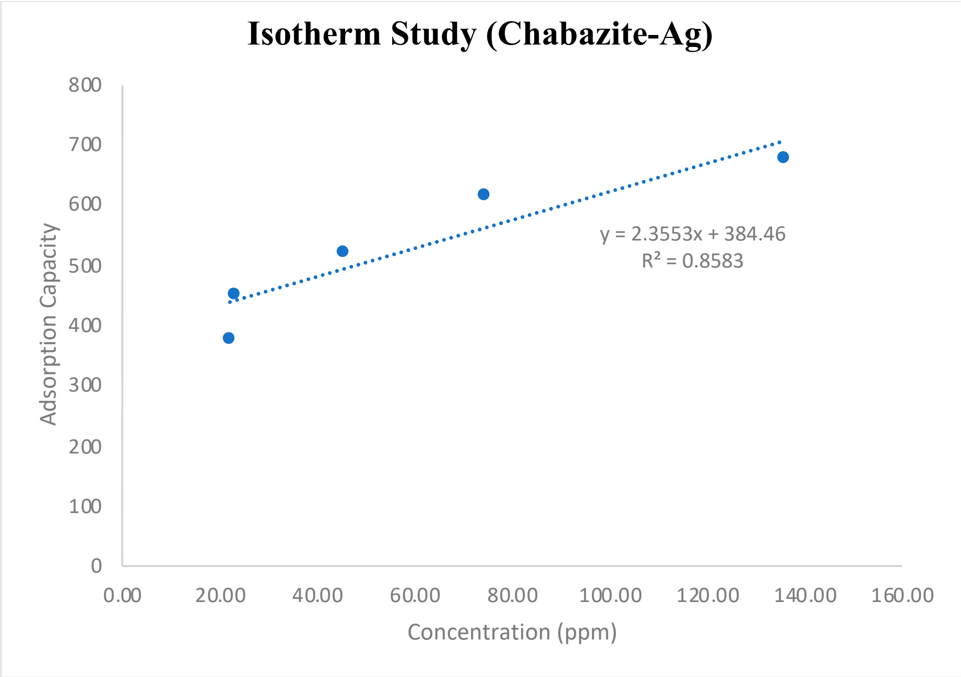

Figure S10. Isotherm Study for chabazite-Ag Measured with UV-Vis Spectrophotometer at 462 nm.

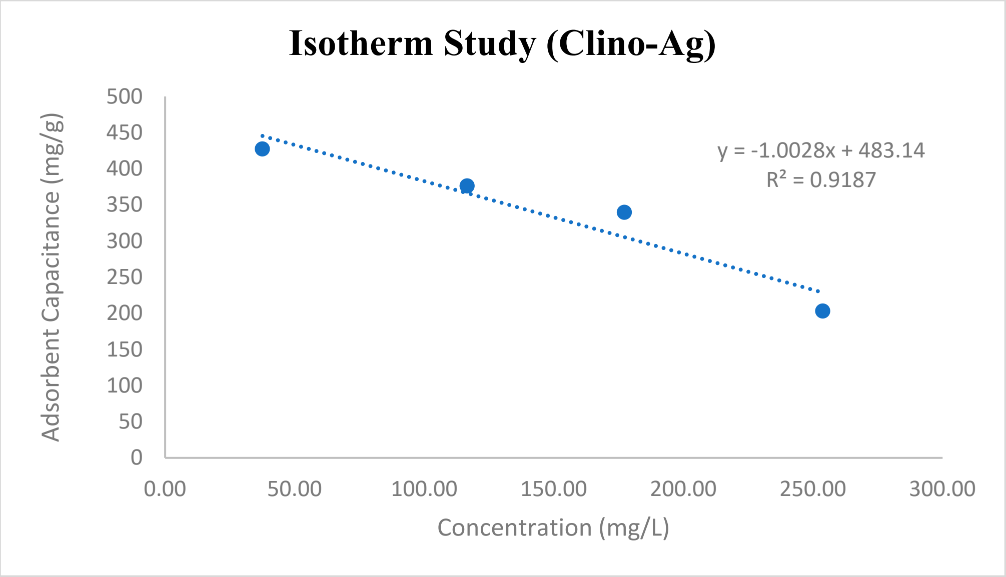

Figure S11. Isotherm Study for clinoptilolite-Ag Measured with UV-Vis Spectrophotometer at 462 nm.

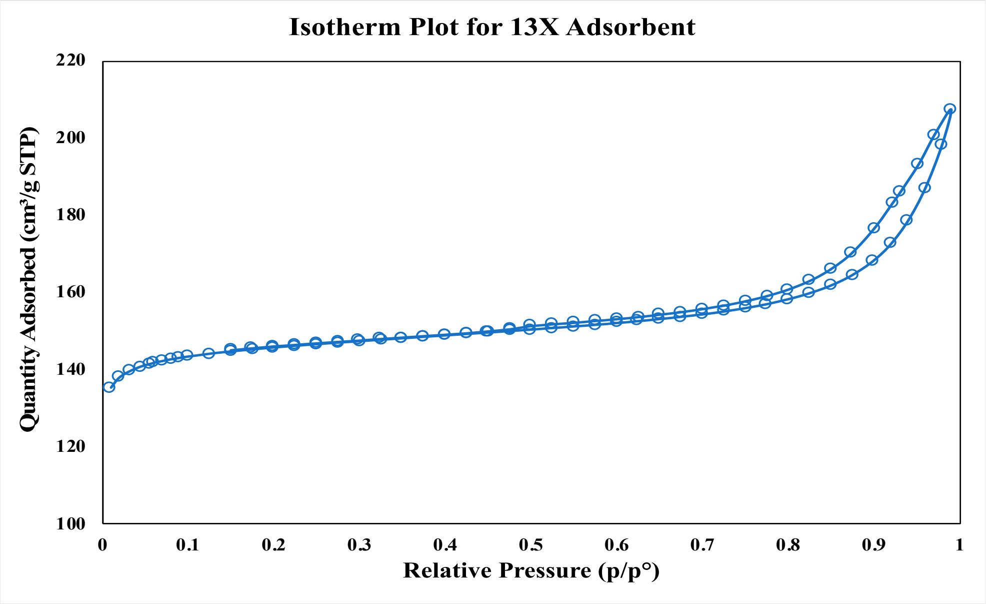

Figure S12. Nitrogen adsorption–desorption isotherm of 13X zeolite Adsorbent.

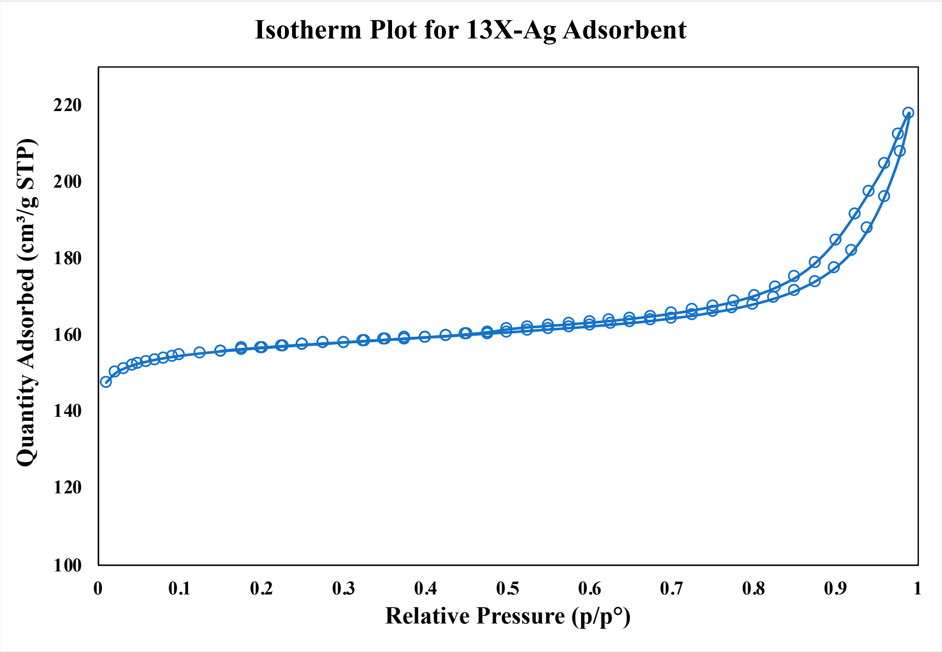

Figure S13. Nitrogen adsorption–desorption isotherm of 13X-Ag zeolite Adsorbent.

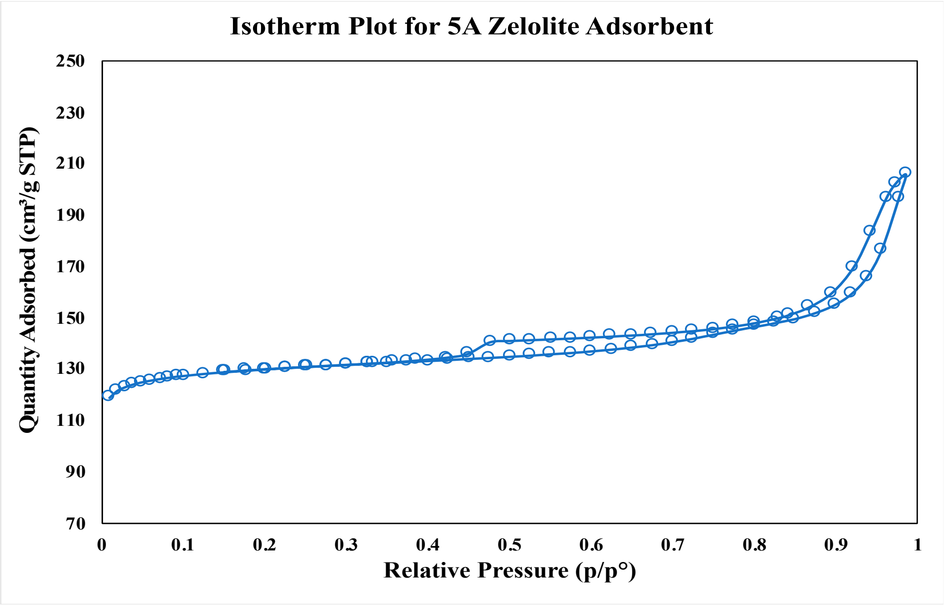

Figure S14. Nitrogen adsorption–desorption isotherm of 5A zeolite Adsorbent.

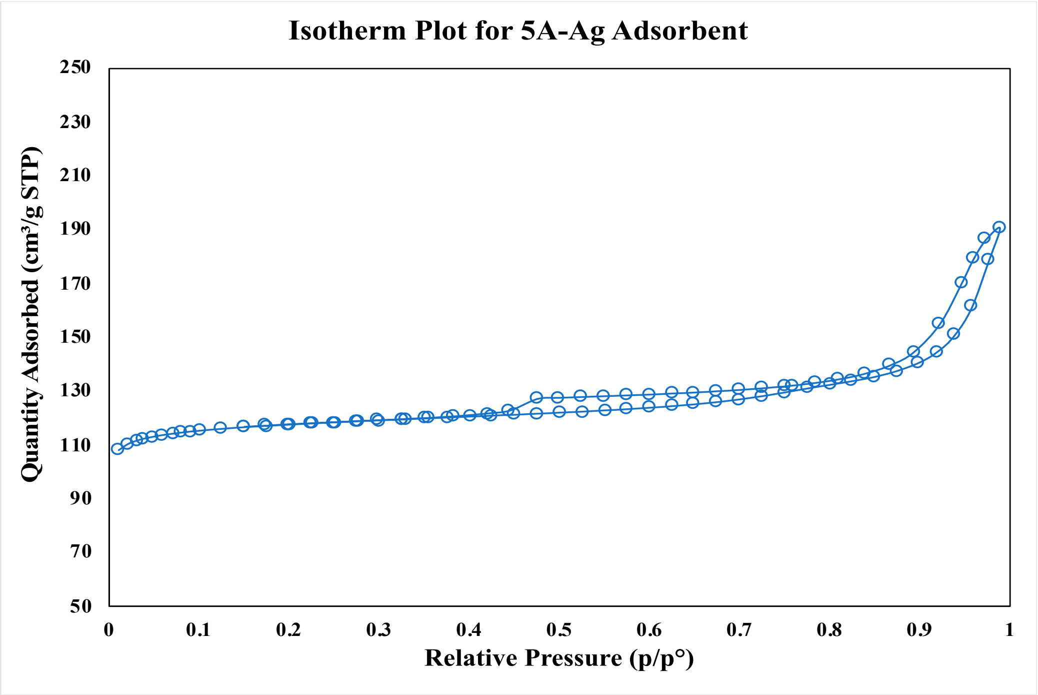

Figure S15. Nitrogen adsorption–desorption isotherm of 5A-Ag zeolite Adsorbent.

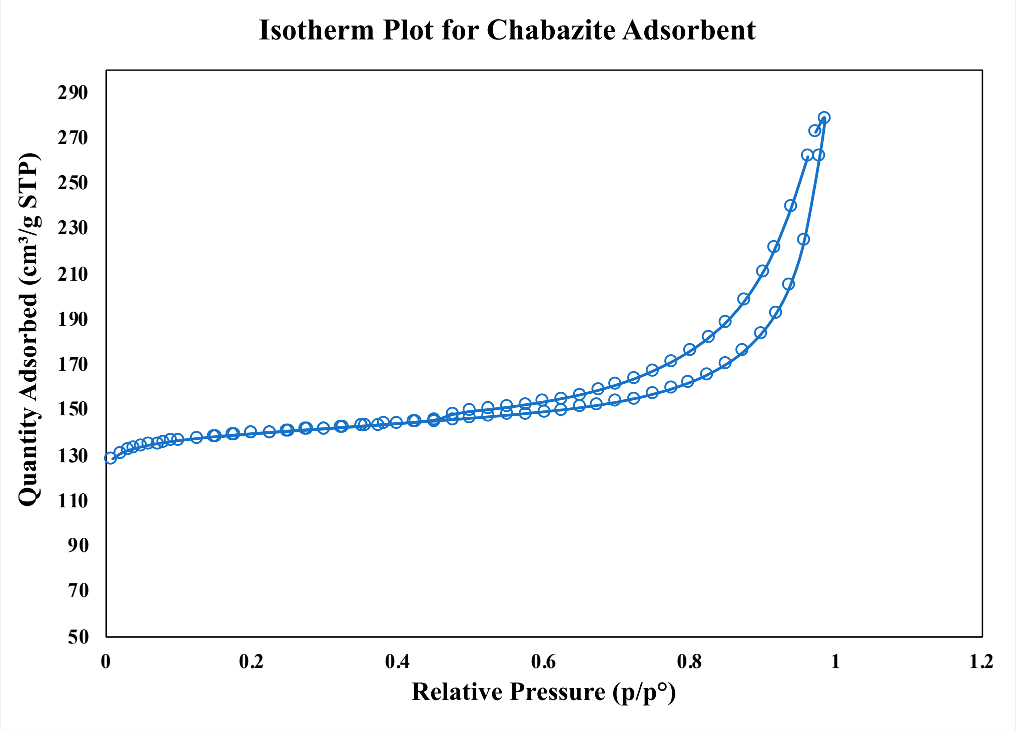

Figure S16. Nitrogen adsorption–desorption isotherm of Chabazite zeolite Adsorbent.

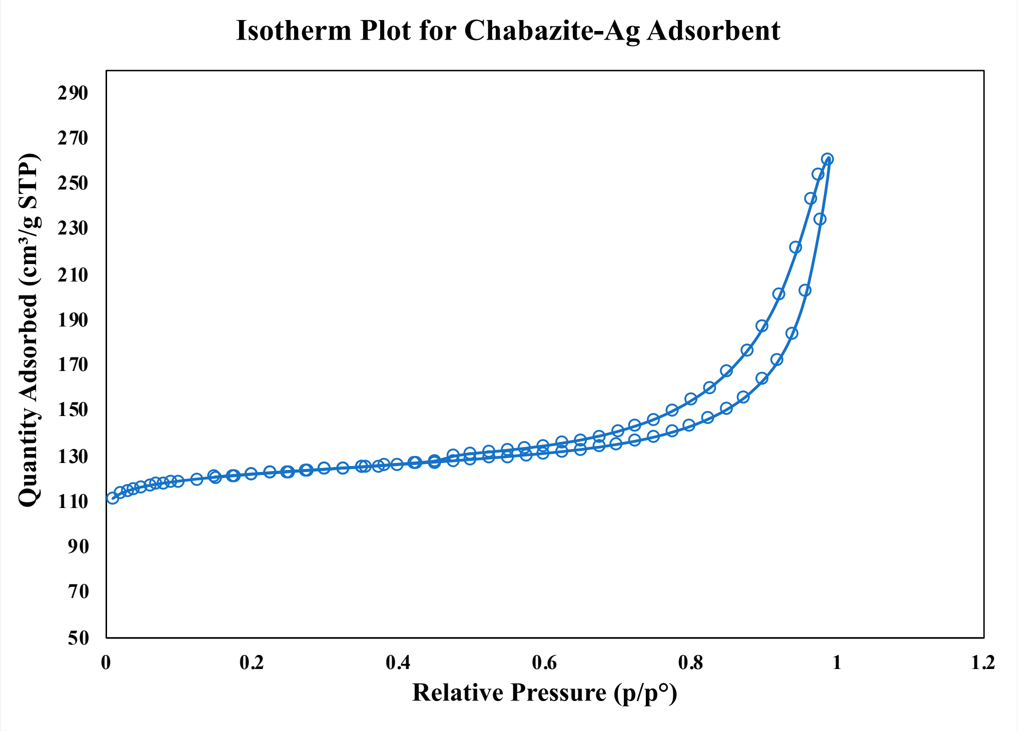

Figure S17. Nitrogen adsorption–desorption isotherm of Chabazite -Ag zeolite Adsorbent.

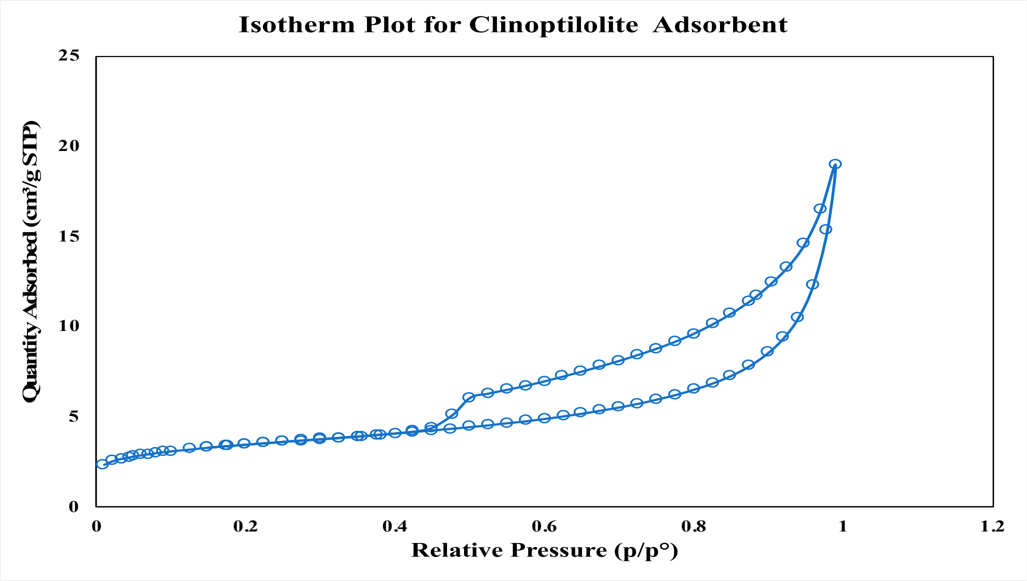

Figure S18. Nitrogen adsorption–desorption isotherm of Clinoptilolite zeolite Adsorbent.

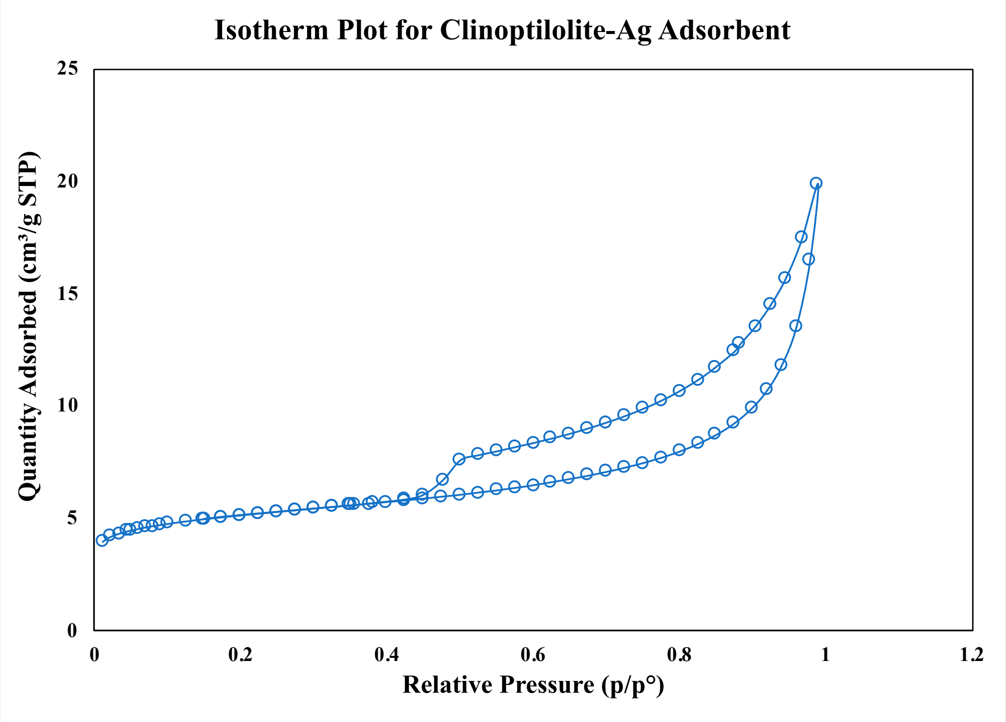

Figure S19. Nitrogen adsorption–desorption isotherm of Clinoptilolite -Ag zeolite Adsorbent.

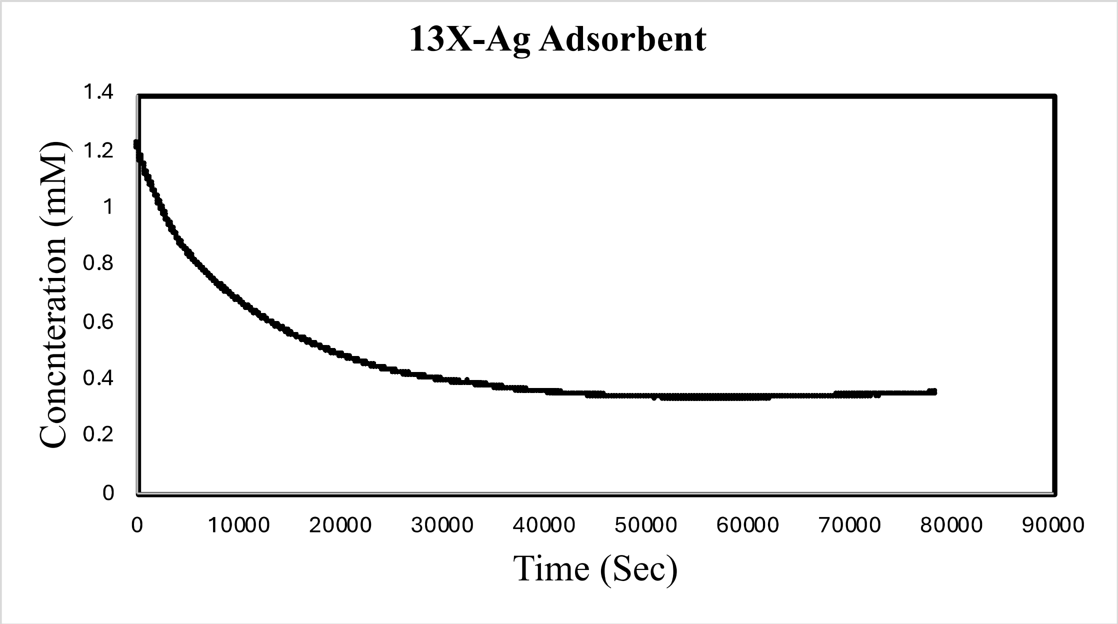

Figure S20. Adsorption kinetics of iodine on 13X-Ag adsorbent.

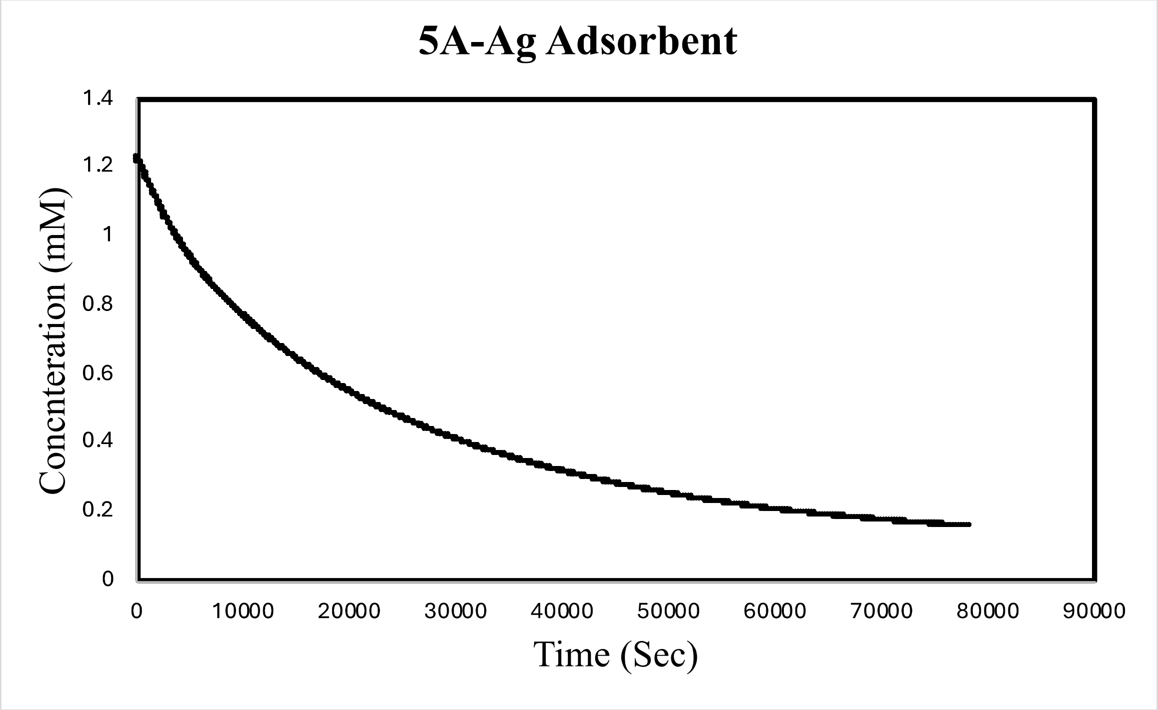

Figure S21. Adsorption kinetics of iodine on 15A-Ag adsorbent.

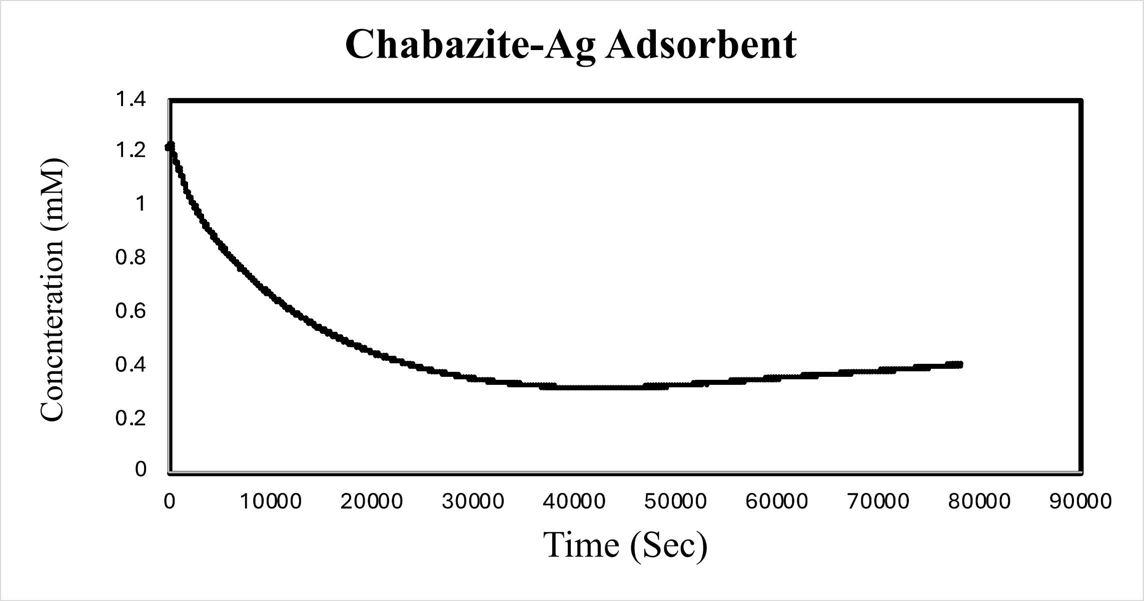

Figure S22. Adsorption kinetics of iodine on Chabazite-Ag adsorbent.

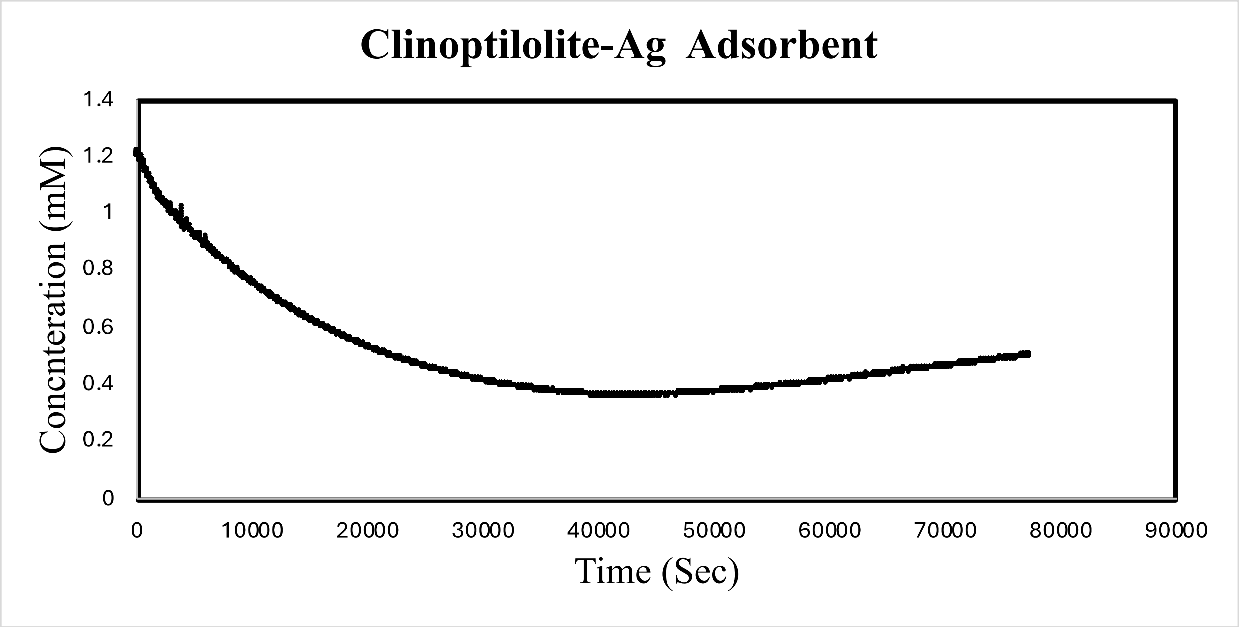

Figure S23. Adsorption kinetics of iodine on Clinoptilolite-Ag adsorbent.

Table S1. Element weight percentages by EDS for 13X and 13X-Ag adsorbent.

13X		13X-Ag	
**Element**	**wt %**	**Element**	**wt %**
**C**	6.09	**C**	4.4
**O**	51.2	**O**	47.12
**Na**	10.07	**Na**	6.56
**Mg**	0.81	**Mg**	0.8
**Al**	11.59	**Al**	14.92
**Si**	19.31	**Si**	17.77
**K**	0.33	**Ag**	7.98
**Ca**	0.55	**Fe**	0.45

Table S2. Element weight percentages by EDS for 5A and 5A-Ag adsorbent.

5A		5A-Ag	
**Element**	**wt %**	**Element**	**wt %**
**C**	2.36	**C**	17.4
**O**	52.62	**O**	43.77
**Na**	1.79	**Na**	0.76
**Mg**	1.27	**Mg**	0.95
**Al**	13.63	**Al**	10.38
**Si**	18.18	**Si**	11.61
**Ca**	8.81	**Ca**	4.41
**Cl**	0.53	**Cl**	0.56
**Fe**	0.82	**Fe**	0.47
-	-	**Ag**	9.68

Table S3. Element weight percentages by EDS for Chabazite and Chabazite-Ag adsorbent.

Chabazite		Chabazite-Ag	
**Element**	**wt %**	**Element**	**wt %**
**C**	7.33	**C**	17.85
**O**	49.28	**O**	44.58
**Na**	5.96	**Na**	3.15
**Mg**	0.36	**Mg**	0.32
**Al**	7.64	**Al**	5.1
**Si**	24.69	**Si**	16.65
**Cl**	0.26	**Cl**	1.24
**k**	0.80	**K**	0.67
**ca**	0.48	**Fe**	2.08
**Fe**	2.85	**Ag**	8.36

Table S4. Element weight percentages by EDS for Clinoptilolite and Clinoptilolite -Ag adsorbent.

Clinoptilolite		Clinoptilolite-Ag	
**Element**	**wt %**	**Element**	**wt %**
**C**	13.13	**C**	11.98
**O**	49.45	**O**	49.86
**Na**	2.3	**Na**	1.04
**Mg**	1.11	**Mg**	0.51
**Al**	4.47	**Al**	4.25
**Si**	24.52	**Si**	22.16
**S**	0.22	**K**	1.93
**Cl**	0.13	**Fe**	1.11
**K**	2.34	**Ag**	7.17
**Ca**	0.64	**-**	**-**
**Fe**	1.71	**-**	**-**
